# The Effect of Nanoconfinement on Deliquescence of CuCl_2_ Is Stronger than on Hydration

**DOI:** 10.1021/acs.cgd.2c00821

**Published:** 2023-02-10

**Authors:** Michaela C. Eberbach, Henk P. Huinink, Aleksandr I. Shkatulov, Hartmut R. Fischer, Olaf C. G. Adan

**Affiliations:** † Eindhoven University of Technology, Den Dolech 2, 5600 MB Eindhoven, The Netherlands; ‡ EIRES, Horsten 1, 5612 AX Eindhoven, The Netherlands; ⊥ German Aerospace Center (DLR), Pfaffenwaldring 38-40, 70569 Stuttgart, Germany; § TNO Materials Solutions, High Tech Campus 25, 5656 AE Eindhoven, The Netherlands; ∥ Cellcius BV, Horsten 1, 5612 AX Eindhoven, The Netherlands

## Abstract

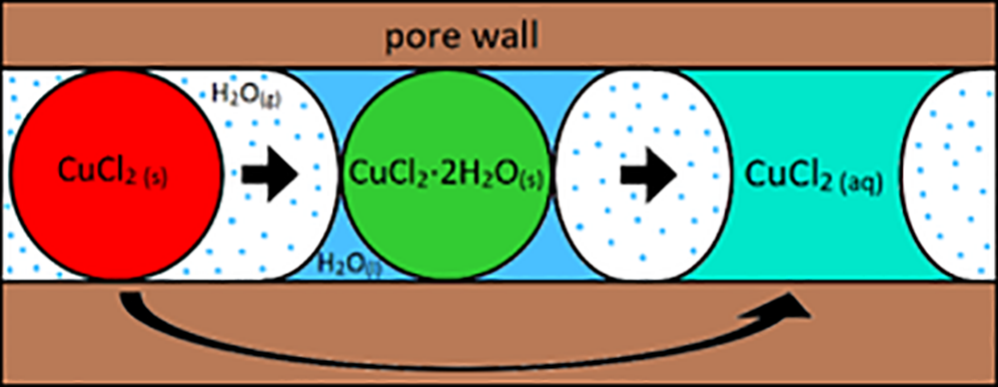

The hydration of salts has gained particular interest within the frame of thermochemical energy storage. Most salt hydrates expand when absorbing water and shrink when desorbing, which decreases the macroscopic stability of salt particles. In addition, the salt particle stability can be compromised by a transition to an aqueous salt solution, called deliquescence. The deliquescence often leads to a conglomeration of the salt particles, which can block the mass and heat flow through a reactor. One way of macroscopically stabilizing the salt concerning expansion, shrinkage, and conglomeration is the confinement inside a porous material. To study the effect of nanoconfinement, composites of CuCl_2_ and mesoporous silica (pore size 2.5–11 nm) were prepared. Study of sorption equilibrium showed that the pore size had little or no effect on the onsets of (de)hydration phase transition of the CuCl_2_ inside the silica gel pores. At the same time, isothermal measurements showed a significant lowering of the deliquescence onset in water vapor pressure. The lowering of the deliquescence onset leads to its overlap with hydration transition for the smallest pores (<3.8 nm). A theoretical consideration of the described effects is given in the framework of nucleation theory.

## Abstract 2

This paper reports an investigation of hydration and deliquescence phase transition of CuCl_2_ under confinement with different pore diameters.

## Introduction

Global energy consumption increases every year^1^ and is still mainly produced by fossil fuels, such as coal, oil, and gas, which is concerning due to climate change. There is already a shift in the production of energy toward renewable energy sources to reduce the amount of CO_2_: wind and solar energy production. However, there is a mismatch between energy supply and demand; for example, solar energy and heat production are high during the sunny days, in summer, while the demand is low.^2^ A majority of the energy, in residential homes, is used for heating;^3^ therefore, in winter the heating demand is high while the supply of thermal energy is low. A solution for the mismatch problem is thermal energy storage (TES), which can be used to heat during summer and release the stored heat during winter. There are three types of TES: sensible, latent, and thermochemical energy storage (TCES).^4,5^ Compared to sensible and latent heat storage, TCES can store heat loss-free,^5,6^ which is necessary for long-term or seasonal heat storage.

**Figure 1:**
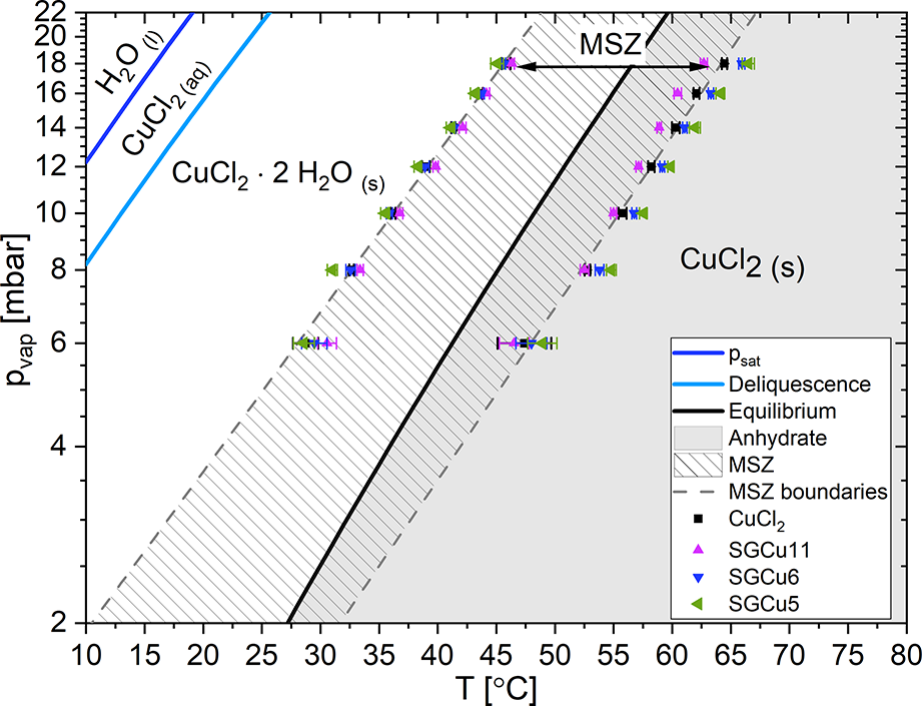
Here, the phase behavior of CuCl_2_ as a function of the water vapor pressure *p*_vap_ and the temperature *T* is shown. The condensation line (*p*_sat_), deliquescence onset line, (de-)hydration equilibrium line, and metastable zone (MSZ) with its boundaries are depicted.^9^ The (de)hydration onset points of pure CuCl_2_ (black squares) and the composites SGCu11 (purple triangle up), SGCu6 (blue triangle down), and SGCu5 (green triangle left). The MSZ boundaries as fitted from the results of the pure CuCl_2_ are denoted as dashed lines.

For low-temperature TCES, salt hydrates and porous media are being investigated. The porous media adsorb large amounts of water molecules due to their high specific surface.^7,8^ Salt hydrates build in the water molecules from the vapor phase into their crystal structure, which is called hydration. The hydration of a salt is an exothermic solid–solid phase transition^5^ involving (water) vapor absorption, which can be reversed via an endothermic reaction by heating the crystals. This phase transition happens at a certain water vapor pressure (*p*_vap_) and temperature (*T*) depending on the transition. The equilibrium between hydrates is reached when the conditions around the salt particles are set to water vapor pressure and temperature values such that the hydration and dehydration are happening at the same rate. Such an equilibrium between two states can be found in a pT-phase diagram as a line separating the two phases (Figure 1). This type of (water) vapor sorption is described as monovariant and is detailed in ref (18). Many salts have slow kinetics in a region around the equilibrium line of the solid–solid phase transition between hydration states, which is called the metastable zone of the (de)hydration reaction (MSZ).^9−11^ The MSZ hinders the (de)hydration transition due to a nucleation barrier.^10,11^ Dehydration/hydration of salt hydrates theoretically provide very high energy storage density up to 3.6 GJ/m^3^.^4,5,12−14^ However, salts expand and shrink during the charging and discharging cycles, due to the built-in release water molecules in the salt hydrate crystal structure.^15,16^ Moreover, conglomeration and deliquescence of the salt particles can be a problem over many cycles, since this could block the transport of the water vapor through the storage unit. Contrary to the salt hydrates, porous materials are usually hydrothermally stable over many cycles, because they generally do not change their solid structure. There are some exceptions like metal–organic frameworks (MOFs). Usually, porous materials have lower energy storage densities. Additionally, the type of sorption done by porous materials is described as bivariant, because vapor can be adsorbed continuously with an increase in vapor pressure and/or decrease in temperature instead of a sharp transition like with salt hydrates.^18^

A solution for the disadvantages of these two materials can be offered by a combination of the two TCES types - composite of salt in porous matrix (CSPM).^5,17,18^ The energy storage capacity of the porous matrix, with its water adsorption, could be increased through the stronger water absorption of the salt hydrate. Simultaneously, the salt may be stabilized inside the pore structure, where it can expand and shrink during cycling, while the matrix keeps the macroscopic shape constant.^19^ Additionally, the deliquescence transition can be used as an extra phase transition to store and release heat, because the capillary forces of the pore system can hold the salt solution inside the porous matrix.^20^

Many different CSPMs have been investigated. Often silicas,^21^ zeolites,^22,23^ and porous clays, like vermiculite, were used as matrices to host CaCl_2_, LiBr, LiCl, or magnesium salts^5,24−26^ among many other combinations of matrices and salts. These composites have energy densities ranging from 0.18 to 1.08 GJ/m^3^, which are lower than for the pure salt due to the volume of the matrix material and the void space inside the pores to allow the mass transport of the gas phase. Also, hydrothermally stable metal–organic frameworks (MOFs),^27^ aerogels,^24^ carbon based materials,^5^ and porous glass^28^ were tested as matrices and different salts like SrBr_2_, copper, potassium, and sodium salt hydrates were explored as impregnates^24,29,30^ to improve the CSPM storage materials. Additionally, synthesized matrices such as polymers^31,32^ and silicone foams^33,34^ were tested. Thereby, it was found that CSPM has generally better sorption kinetics than pure salt.^15,29,35^ It was also determined that a higher salt content in the composite results in higher water uptake and energy densities.^27,30^ However, a too-high salt content could block pores off, mainly in microporous materials.^36^

The studies about the hydration transition with salt in composites were mainly focused on finding good heat storage materials and hence composites with high water uptakes and energy storage densities. However, the impact of confining the salt in a porous matrix on the involved transitions is not fully understood. The salt in CSPMs could have different interactions with water vapor, because of the added interface with the pore walls, which results in a nonlinear combination of the matrix and salt sorption properties.^37^ So, the confinement of the salt inside a porous matrix could change the sorption properties of the salt hydrate. On one hand, the pores adsorb water vapor, which could enhance the absorption speed of the salt. On the other hand, the limitation of the crystal size by the pore size could influence the hydration and deliquescence transitions through increased surface effects. The effect of confinement on the deliquescence transition was already studied for the simple salts of NaCl and LiCl in mesoporous silica materials in ref (39) and ref (40). Here, it was shown that the onset of deliquescence for NaCl and LiCl shifts to lower water vapor pressures in isotherms when confined inside mesoporous silicas due to the concave curvatures of the water–gas interface inside these pores.

The goal of this research is to investigate these effects of confinement on the sorption properties of a model salt hydrate CuCl_2_ inside the pores of amorphous silica gel (SG) with nanometer-sized pores. CuCl_2_ was chosen as a model component for its single hydration transition, the metastable zone (MSZ), and the deliquescence point^9,38^ such that there is a clear distinction between hydration and deliquescence transitions. In this way, the two transitions could be studied separately. Changes in the width of the MSZ were investigated with TGA measurements. The impact of the confinement on the crystalline structure was studied with PXRD. The deliquescence onsets of the different composites were studied via DVS measurements to compare the onsets of CuCl_2_ in confinement to similar studies in the literature on other salts.^39,40^

During the following text, the phrase (de)hydration transition is used to describe the solid–solid phase transition between hydrates through building in or removing water molecules in the crystalline structure. Additionally, the terms anhydrate and (di)hydrate are going to be used to label the two different solid crystalline phases of CuCl_2_, which contain either no water molecules (only CuCl_2_) or two moles of H_2_O per mole of CuCl_2_ (CuCl_2_·2H_2_O). Subsequently, a complete transition is defined as either the removal of all water molecules in the salt crystal to derive the anhydrate (dehydration) or the build-in of whole 2 molecules of H_2_O for each ion pair of CuCl_2_ throughout the full sample to obtain the dihydrate (hydration).

## Theory

**Figure 2:**
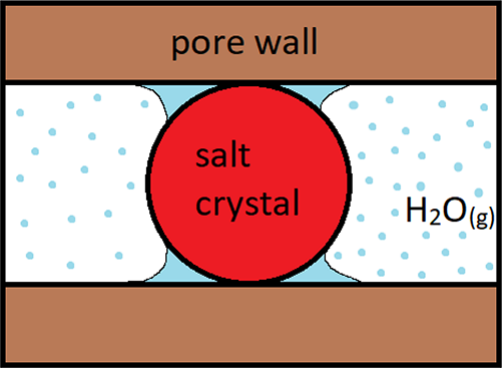
Schematic depiction of a spherical salt crystal confined inside a pore structure in contact with a gas phase, which carries water vapor, and liquid water, which condensates from the water vapor.

Confinement of salt hydrates could change their sorption behavior in two ways: (a) increase the surface-to-volume ratio (S/V) of a crystal and hence the surface effects, and (b) sorption due to capillary condensation inside the pores. A schematic representation of such a composite system is given in Figure 2. Three main parameters can describe the sorption behavior of a salt hydrate, which could be affected: (1) thermodynamics of the CuCl_2_–CuCl_2_·2H_2_O transition, (2) the MSZ width of this (de)hydration transition,^9^ and (3) the deliquescence onset. The MSZ of the (de)hydration solid–solid phase transition is a kinetically hindered region around the equilibrium line as described in the Introduction.^9−11^

In the subsequent sections, we will discuss the influence of nanometer-sized, mesoporous confinement on the (de)hydration solid–solid phase transition, metastable zone width of this transition, and deliquescence onset of the salt hydrate CuCl_2_.

### The Hydration Equilibrium in Nanometer-Size Confinement

The equilibrium line of the anhydrate-dihydrate (0–2) solid–solid phase transition of copper(II) chloride (CuCl_2_) can be seen in Figure 1 as the thick black line in between the anhydrous CuCl_2_ and the dihydrate CuCl_2_·2H_2_O. At low water vapor pressures and high temperatures than the equilibrium conditions, the salt is in the anhydrous state. At higher water vapor pressures and lower temperatures, the salt is in the hydrated state. This solid–solid phase transition can be described by the reaction equation:
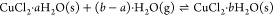
1*a* describes the number of water molecules per salt molecule in the lower hydrated phase and *b* the number of water molecules per molecule of salt in the higher hydrated phase. In the case of CuCl_2_, the *a* and *b* are equal to 0 and 2 for the anhydrate and dihydrate of the salt.

The Gibbs free energy of this reaction equals:^9^
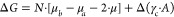
2Here, Δ*G* [J] is the Gibbs free energy, *N* [−] is the number of neutral ion pairs of CuCl_2_, μ_*i*_ [J] is the chemical potential per molecule/neutral ion pair of the different salt phases, μ [J] is the chemical potential per molecule of the water vapor, γ_c_ [J/m^2^] is the specific interfacial surface energy of the solid salt crystal with the vapor phase, and *A* [m^2^] is the surface area of the salt crystal. From this and the derivation given in ref (41), the following can be obtained:
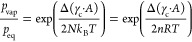
3The *p*_vap_ [mbar] represents the partial water vapor pressure, *p*_eq_ [mbar] is the water vapor pressure at the equilibrium line, *k*_B_ [J/K] is the Boltzmann constant, *T* [K] is the absolute temperature, *R* [J/mol K] is the ideal gas constant, and *n* [mol] is the number of moles of CuCl_2_. With this equation, *p*_vap_, at which the thermodynamic hydration transition happens, can be described as an affirmation of the interfacial properties and the crystal size.

The surface/interface energy (Δ(γ_c_·*A*)) plays a more important role for smaller salt crystals than larger ones because there is a larger surface-to-volume ratio. To investigate the effect of this crystal size, the thermodynamic hydration onset for spherical salt crystals with different diameters was calculated. Because the Δγ_c_ for the salt crystal is not known, the calculations were done by using Δ*A* between the anhydrate and dihydrate, while Δγ_c_ was tested with different values (0.001–0.120 J/m^2^) and their negative counterparts. The values for Δγ_c_ were chosen in a wide range around the suspected range found in ref (9) for the hydration transition of spherical crystals. Thereby, it was found that the difference between *p*_vap_/*p*_eq_ and 1 increased with larger values of γ and smaller crystal sizes. A graphical representation of these relations can be found in the SI.

### The Metastable Zone Boundaries in Nanometer-Size Confinement

Many salts do not start (de)hydrating immediately for their solid–solid phase transition when the equilibrium line is crossed during a decrease or increase in temperature or water vapor pressure, but at a certain temperature or water vapor pressure difference with the equilibrium line or after an induction time, as was described in the introduction as a metastable zone (MSZ). CuCl_2_ is one of these salts that have an MSZ, which is shown in Figure 1 as the hatched area with the dashed lines as its borders.

The metastability is a consequence of kinetic hindrance and nuclei forming only immediately at the boundaries and outside the MSZ. At the MSZ boundary the critical size drops dramatically, allowing nearly barrierless nucleation outsize MSZ.^9,10^ The Gibbs free energy, eq 2, can be used to determine the critical nucleus size for the phase transition at different points in the phase diagram. According to classical nucleation theory, the critical size of a spherical nucleus can be estimated:^42^
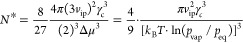
4*v*_ip_ [m^3^] represents the volume of a neutral ion pair of the anhydrous phase and γ_c_ is the specific interfacial surface energy of the anhydrate, which was the starting phase for the Gibbs free energy (eq 2). In the first part of this equation, the phase diagram data (*T* and *p*_vap_) are not direct variables of the critical nucleus. So, the Δμ term can be replaced by temperature and pressure terms, like
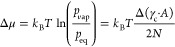
5By doing so, the critical nucleus size N* can be found at every point in the dihydrate part of the phase diagram using the parameters *T* and *p*_vap_. By using the value of *p*_vap_/*p*_eq_ at the MSZ hydration boundary,^9^ the critical nucleus size at this line can be determined. In the range of temperatures of <50 °C and Δγ < 0.040 J/m^2^ the size of critical nuclei does not exceed 2.5 nm. As long as the nucleus diameter is smaller than the pore diameter, no changes in the MSZ are expected. However, if the diameter of the nucleus would be larger than the pore diameter, a hindrance to the solid–solid hydration phase transition is expected, since the nucleus is impossible to form under these conditions. Smaller nuclei, that can be formed in the limited space inside the pores are expected at higher supersaturation/driving force, such as a larger difference in temperature or vapor pressure to the equilibrium conditions, resulting in a widening of the MSZ. This is expected for the tested nanometer pore diameters, especially the smallest one of 2.5 nm, in the case of Δγ_c_ values above 0.04 J/m^2^. A graphical representation of these expected results is given in the SI.

### The Deliquescence Onset in Nanometer-size Confinement

The deliquescence onset of CuCl_2_ is visualized in Figure 1 by the thick light-blue line, which is lower than the saturation water vapor pressure (thick dark-blue line). Here we consider the capillary condensation in cylindrical pores. The capillary condensation in cylindrical pores is described by the Kelvin equation:^43,44^
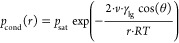
6Here, the *p*_cond_(*r*) [mbar] is the water vapor pressure at which capillary condensation starts in pores with radius *r*, *p*_sat_ [mbar] is the saturation water vapor pressure outside a pore system, *v* [m^3^/mol] is the molar volume of water in the liquid phase, γ_lg_ [J/m^2^] stands for the surface tension between the liquid and vapor phase, θ [°] stands for the contact angle of the liquid phase with the solid and vapor phase, and *r* [m] stands for the radius of the pores.

When the influence of crystal size on the solubility is neglected, the Kelvin equation can be used to estimate a trend of a shifted deliquescence onset toward lower water vapor pressures of salt inside nanosized pores:
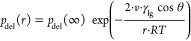
7Here, *p*_del_(*r*) [mbar] represents the water vapor pressure of the deliquescence onset inside pores with radius *r*, and *p*_del_(*∞*) [mbar] is the deliquescence onset water vapor pressure of the bulk salt. In this equation, γ_lg_ describes the surface tension between the liquid and gas phases. Since γ_lg_ for the saturated CuCl_2_ solution is unknown, γ_lg_ for water is used for an estimation. This shows that the condensation points of the water vapor and the deliquescence onset of salt are expected to be at lower water vapor pressures or at higher temperatures when inside smaller pore sizes. These two mechanisms, capillary condensation, and deliquescence, result both in a dissolution of the salt inside the pores but are initiated in different ways: the capillary condensation by the pores in the porous material and the deliquescence onset through absorption of water vapor in the salt to form a saturated solution. From these estimations, the deliquescence onset of the confined salt is expected to always be notably lower than the onset of the capillary condensation with the same pore size. Graphs of these results inside the phase diagram are shown in the SI.

A simplified thermodynamic model for the deliquescence of nanosized particles, which takes the solubility of the salt crystal into account, was developed by Mirabel et al.^45^ This model indicates that the solubility of small soluble crystals in a vapor of solvent increases with decreasing size, due to the increased contribution of the surface energy. Through this increased solubility, the deliquescence onset is lowered even further. Other calculations and even first measurements were done on the simple salts NaCl and LiCl in different silica materials with mesopores.^39,40^ There, it was found, that the deliquescence onset of these salts in confinement was shifted to lower water vapor pressures compared to the bulk salt. The same trend is expected in this study for the CuCl_2_ salt confined in the mesoporous silica gels.

## Experimental Section

### Starting Materials

Mesoporous, amorphous SGs were used with different pore diameters, which will be called SG11, SG6, SG5, SG4, SG3, and SG2 because of the rounded measured average pore diameters. The SG4 was ordered from Fisher Scientific in the form of 40 Å pore size, general purpose grade, while all other SGs were ordered from Sigma-Aldrich in the form of pore size 150 Å Davisil grade 643, 90 Å high-purity grade, pore size 60 Å Davisil grade 635, pore size 30 Å Davisil grade 923, and pore size 22 Å Davisil grade 12, and all of them were used without any treatment. As the salt inside the CSPM, copper(II) dichloride (CuCl_2_) was ordered from Sigma-Aldrich in the form of the dihydrate (CuCl_2_·2H_2_O, ACS reagent, ≥99.0%). For the use as a bulk salt reference, CuCl_2_·2H_2_O was ground in a mortar and sieved so that the salt always had a particle size between 50 and 164 μm. For the formation of a saturated solution, 9.5 g of this dihydrate was dissolved in 10 mL of demineralized water, which was used to impregnate the silica gels to create the CSPMs.

### CSPM Preparation by Impregnation

For the impregnation of the salt CuCl_2_ into the pore structure of different SGs, a dry incipient or incipient wetness method was used, as described in refs (24, 29, 36, 46, and 47) First, the SGs were dried in an oven at 160 °C overnight. The dried SGs were mixed with a saturated aqueous solution of CuCl_2_ to fit the accessible pore volume. The components were mixed until the solution was fully adsorbed into the pores of the silica gels. The composites were then dried again overnight in an oven at 160 °C. Through weighing the samples at various steps in the procedure, the masses (*m*) of the two components, the salt content (ϕ), and the weight ratio between the silica gel and the salt could be determined. Here, the salt content was calculated as
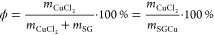
8Compositions of the prepared composites are given in Table 1 with the names SGCu11, SGCu6, SGCu5, SGCu4, SGCu3, and SGCu2 according to the average pore diameter of the used SG. The given salt content for the different composites was the amount reached with one-time impregnation with saturated solution, as described above. Higher salt content could be achieved with multiple impregnations; however, the possibility arises that salt crystals will be formed on the outer surface of the silica gel particles (see Supporting Information (SI)) and/or that (large) parts of the pore systems will be blocked by salt crystals hindering mass and heat transfer. Therefore, the sample preparation was limited to one single impregnation step for this study.

**Table 1:** Weight Ratios Are Shown of the Different Composites Made from Silica Gels with an Average Pore Diameter (*d*_avg_) in the Nanometer Range and a Saturated Solution of CuCl_2_. Below, the Calculated Salt Content (ϕ) and Weight Ratio between the Two Components are given. Here, *m* Stands for Mass in [g].

composite name	pore *d*_avg_ silica [nm]	ϕ wt %	*m*(CuCl_2_)/*m*(silica) [g/g]
SGCu11	11.0	40.03	0.668
SGCu6	6.0	31.90	0.477
SGCu5	5.8	31.38	0.457
SGCu4	3.8	20.41	0.256
SGCu3	3.3	14.81	0.174
SGCu2	2.5	14.43	0.169

### Pore Volume, Pore Size, and Pore Size Distribution

The pore structure of the samples was investigated by N_2_ adsorption and desorption. First, the samples were prepared by degassing them in the preparation station at 150 °C with N_2_ flow overnight (16 h). Both adsorption and desorption measurements were done at 77 K with pressure range *p*/*p*^0^ = 0–0.998 in a Micromeritics Gemini VII. The Barrett–Joyner–Halenda (BJH) method^48^ was used to calculate the average pore diameter, and the Brunauer–Emmett–Teller (BET) theory^49^ was used to determine the surface area in m^2^ per g of sample and the maximum N_2_ adsorption volume to estimate the accessible pore volume.

### Isobaric TGA - Finding MSZ Widths

Isobaric water sorption and desorption of the different SG and the composites were investigated using the two TGA devices Mettler Toledo TGA/SDTA851e and Mettler Toledo TGA/DSC 3+ to prevent measurement artifacts from one single machine. The two TGA setups from Mettler Toledo were used together with a home-build or a Cellkraft humidifier. The sample was located in both devices on a balance arm inside the oven capable of operating at *T* = 25–1000 °C, which had an accuracy of ±1 μg.

Both machines had an inlet for gas flows, which were connected to humidifiers. The home-build humidifier operated at 18 °C and mixed a dry (0% RH) and a saturated (100% RH) N_2_ flow to generate a water vapor pressure between 0 and 20 mbar. This home-build device was connected to the TGA/SDTA851e. The second humidifier was a Cellkraft Humidifier P-2 operating at 25 °C, which worked via a feedback loop from an RH sensor at the outlet of the humidifier to generate a water vapor pressure between 0 and 27 mbar in the TGA/DSC 3+. Both devices had a flow rate set to 300 mL/min over the sample inside the TGAs.

The temperatures of both TGAs were calibrated to an accuracy of 0.2 K using the melting points of benzophenone, indium, and zinc,^50^ while the humidifiers were calibrated to an accuracy of ±1 mbar using the gravimetric signal at the deliquescence point of LiCl·H_2_O, CH_3_COOK, K_2_CO_3_·1.5H_2_O, MgCl_2_·6H_2_O and Mg(NO_3_)_2_·6H_2_O at 25 °C and a validity check at higher temperatures (40 and 65 °C) using LiCl·H_2_O.^51^

A temperature (*T*) program was created such that first an isothermal step of a few hours was done, then a *T* ramp was performed at the constant rate between 0.1 and 1 K/min, usually 1 K/min. The lowest *T* was held again for a few hours, after which the *T* was increased again with the same ramp, and the program ended on an isothermal step as in the beginning. Simultaneously, the humidifier was turned on at the wanted water vapor pressure. The isothermal steps at the maximum and minimum *T* of each cycle were done to ensure a complete phase transition. In between these isothermal steps, the temperature ramps were used to determine phase transition onsets and the amount of sorbed water vapor under these conditions. The resulting weight changes from these isobaric measurements were used to calculate a parameter called loading (*L*), which describes the weight change of the sample in [mol H_2_O per mol CuCl_2_]. The loading was calculated similarly to ref (9) using the dry weight at high temperatures of each sample and the salt content given in Table 1 together in
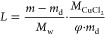
9Hereby, *m* [g] stands for the current mass of the sample, *m*_d_ [g] for the dry sample mass, *M*_w_ [g/mol] for the molecular mass of water (18.01528 g/mol), and  [g/mol] for the molecular mass of copper(II) dichloride anhydrate (134.45 g/mol).

### Powder X-ray Diffraction Analysis in Situ

X-ray diffraction was performed using a Rigaku Mini-Flex diffractometer in continuous scan mode with a divergent slit of 0.625° and a D/teX Ultra2 detector, using Cu K_α_-radiation and K_β_ filter. To identify the crystalline phases of the confined salt hydrates and to observe the phase transitions, powder X-ray diffraction (PXRD) was performed using a high-temperature attachment, called the Anton Paar BTS 500 heating stage, built in the diffractometer and an attached humidifier, which can blow nitrogen with 0–20 mbar water vapor over the sample. The measurement was carried out with Bragg–Brentano geometry at 2θ = 5–75° with step sizes between 0.005 and 0.01° and speed of 1 to 10°/min. The humidifier worked similarly to the home-build humidifier of the TGAs, but the flow rate was set to 800 mL/min, because of larger sample sizes.

Two types of PXRD measurements were performed. First, a scan of the dehydrated composites was performed. This was done at 120 °C and 0 mbar water vapor pressure with the 2θ ranging from 10 to 75°, a step size of 0.005°, and a speed of 3°/min. Each of these scans took 25 min 17 s, and additionally the temperature was held constant for 30 min to ensure complete dehydration.

Second, isobaric in situ measurements were performed using several scans at different temperatures with a constant water vapor pressure turned on. Because it was required to perform 20–30 scans for in situ measurements, the duration of the scans was shortened to reduce the measurement time. Considering that the strongest reflections of the anhydrate and dihydrate lie very close—15.3° and 16.3°^52^—the range of 2θ was shortened from 10–75° to 14–17.4° with a step size of 0.050° and a speed of 10°/min, which changed the recording time of one scan to 1 min 13 s. The isobaric in situ PXRD measurement was executed in the following way: First, the sample was brought to the starting temperature of 120 °C, and then the first scan was recorded. Afterward, the temperature was decreased from 50 to 54 °C to 34–38 °C, depending on the water vapor pressure, in steps of 2 °C. A diffractogram was recorded at each temperature step. Then the diffractogram at the lowest temperature of 33 °C was measured with a subsequent increase in temperature from 46 to 52 °C to 68–74 °C, depending on the water vapor pressure, in steps of 2 °C and end temperature of 80 °C. The start, lowest, and end temperatures were held for 3 h before the reflections were recorded to ensure that the sample was completely transitioned toward its anhydrous or dihydrate phase, while all other temperatures were only held for 1 min before the recording of the reflections. Runs were performed at different water vapor pressures: 10, 12, and 14 mbar.

### Sorption Isotherms - Dynamic Vapor Sorption

Water sorption isotherms were measured with TA Instruments Q5000 SA. This DVS machine controls the temperature (±0.1°*C*) and the relative humidity (RH) (±1%), while the weight of the sample is measured. The RH in this device was calibrated using the deliquescence of NaBr. The sample weight can be measured with an accuracy of 0.01 μg.

The isotherms at 45 °C were measured in steps of increasing humidity. Before an experiment, a dehydration step at 80 °C and 0% RH was performed. For the composites and pure salt, the sorption isotherms were measured with steps in humidity from 0 to around 70% RH. After equilibration at 45 °C, the sample was exposed to the steps of increasing humidity. At each step, the sample was left to equilibrate, which was determined by a change in weight less than 0.005% for 120 min or when the maximum step length of 12 h was reached, which only occurred during phase transitions such as the solid–solid hydration transition or the solid–liquid deliquescence when the weight change is large than the steps without phase transition. By doing so, the hydration and deliquescence onsets of the pure salt and the different composites could be observed through the changes in weight, when water vapor was taken up by the sample. This is a dynamic phenomenon, not a thermodynamic one, which can for example change with different humidity steps or waiting times.

## Results and Discussion

The influence of the SG on the (de)hydration onsets of the salt was studied with TGA measurements. The structure of the salt during water sorption was examined with isobaric XRD in situ experiments. Deliquescence of the salt in the composites was studied by DVS isotherms. The characterizations of the pure SGs are given in the SI.

### Nitrogen Sorption Measurements

The pore structures of the pure silica gels as well as the composites were characterized with N_2_ adsorption and desorption isotherms at 77 K as described in the section above. These isotherms of the pure silica gels are shown in SI. The pore size distributions, obtained with the BJH method from the desorption isotherm and were used to calculate the results in Table 2. From this table, it is visible that the average pore diameters (*d*_*avg*_) of the composites were slightly increased compared to the pure silica gels. This was probably due to the space taken up by the salt crystal, which decreased the pore volume and shifted the pore size distribution slightly. The obtained average pore diameters of the pure silica gels were then used to calculate the capillary condensation point with the Kelvin eq (eq 6).

**Table 2:** Characterization of the Silica Gel Pore Structure, in the Form of Pure Silica Gel and in the Composites, by the Average Pore Diameter (*d*_avg_) in the Nanometer Range, Calculated Using the BJH Method on the Desorption Part of the Isotherm

silica gel name	*d*_avg_ silica [nm]	composite name	*d*_avg_ composite [nm]
SG11	11.0	SGCu11	12.1
SG6	6.0	SGCu6	6.8
SG5	5.7	SGCu5	6.2
SG4	3.8	SGCu4	3.9
SG3	3.3	SGCu3	3.3
SG2	2.5	SGCu2	2.5

### Metastable Zone under Confinement

**Figure 3:**
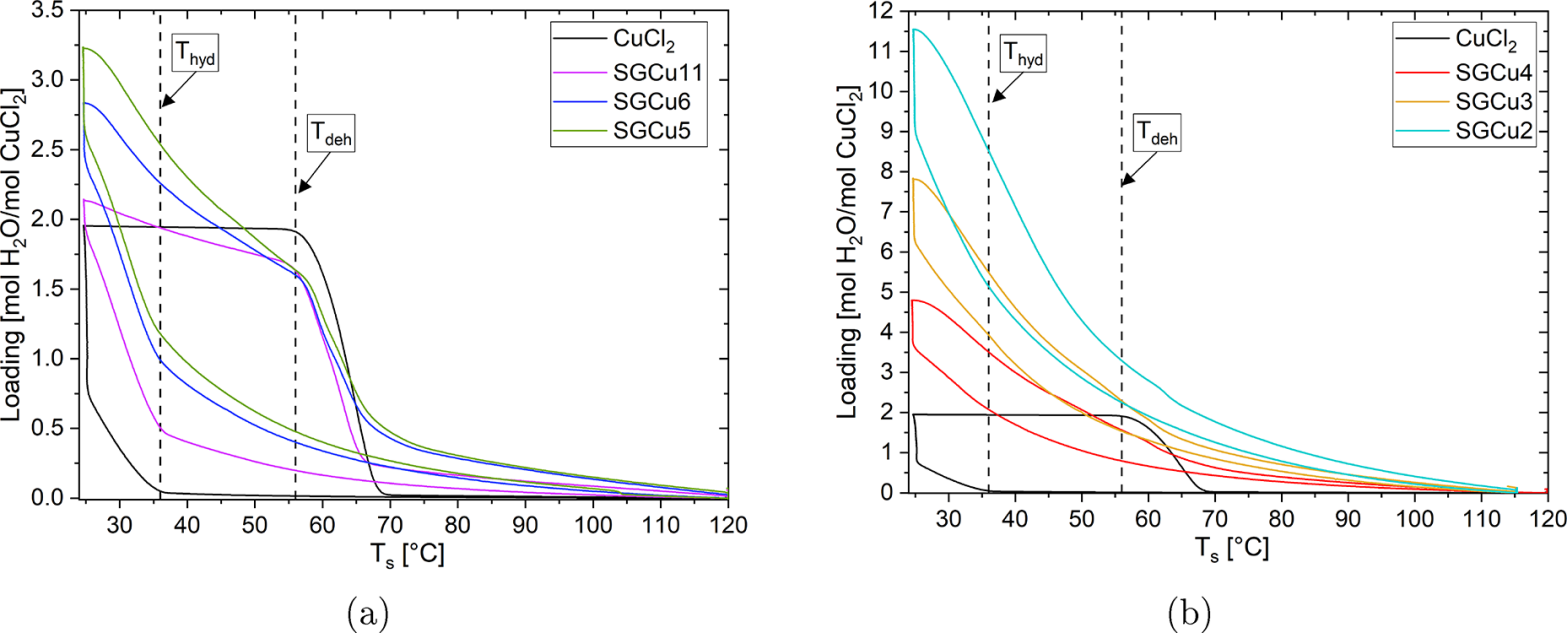
Loading [mol H_2_O/mol CuCl_2_] is given of the water, which was sorbed by salt impregnated silica gel composites as a function of the sample temperature *T*_s_ at a water vapor pressure of 10 mbar in comparison with pure salt are shown: a) SGCu11, SGCu6, and SGCu5 and b) SGCu4, SGCu3, and SGCu2. A temperature change speed of 1 K/min was used, and the dashed lines indicate the hydration and dehydration onsets of the pure CuCl_2_ salt.

First, the sorption of the pure salt and the salt inside the mesoporous SG11 was investigated with an isobaric TGA measurement, at a water vapor pressure of 10 mbar (Figure 3a). The hydration and dehydration onsets were found to be at 36.5 and 55.3 °C, respectively. During this measurement, the pure salt had no or negligible adsorption at a temperature above the hydration onset. Below this temperature, the expected amount of 2 mol of H_2_O per mol of CuCl_2_ was absorbed. During heating of the completely hydrated sample again, this loading remained constant until the dehydration onset, when the salt lost its hydration water again.

The SGCu11 hydrates and dehydrates at the same temperatures as the pure salt. However, before and after these (de)hydration transitions, continuous sorption and desorption are visible. This is due to the adsorption by the SG pores as discussed in the SI. This also explains the higher maximum loading of mol H_2_O per mol CuCl_2_ for the composite.

Subsequently, a series of composites with smaller pore radii were investigated. These composites were subjected to the same measurement conditions (Figure 3). In Figure 3a, the same behavior as with the SGCu11 was observed: similar (de)hydration onsets as bulk CuCl_2_ and continuous ad- and desorption as the SGs. The maximum loading increased hereby with decreasing pore diameter due to the lower salt content as described in Table 1 and the definition of the loading as the sorbed water of the whole sample per amount of salt in the sample. Hence, the silica gel adsorption uptake increases relative to the uptake of the salt and has a stronger influence on the sorption curve with decreasing salt content.

In the case of SGCu4, SGCu3, and SGCu2, the (de)hydration onsets could not be identified. This could indicate that this transition is not visible due to the stronger adsorption of the silica gel or low crystallinity of the salt. The lower salt content for these composites with smaller pore sizes, as described earlier, supports the poor visibility of these onsets. However, if a linear combination of the sorption from the silica gels and the bulk salt with the corresponding salt content is taken of these three composites, the onsets of the (de)hydration are all visible. So, the ambiguity of the onsets in the composites SGCu4-SGCu2 is not expected to come from the low salt content and consequently stronger silica gel adsorption. Subsequently, the smeared onsets could indicate that either the salt cannot form crystalline phases in such small pores, such that no (de)hydration transition is performed anymore, or that the deliquescence is lowered, as discussed formerly in the theory section, to an extent that the hydrated phase is not visible with the TGA measurements for these composites.

Since the (de)hydration onset temperatures are visible for the SGCu11, SGCu6, and SGCu5, these onsets were measured at different water vapor pressures to see if the MSZ boundary is affected by the pore size. Each experiment was performed at 6, 8, 10, 12, 14, 16, and 18 mbar at temperatures varying between 125 and 25 °C with a ramping speed of 1 K/min. The (de)hydration reactions at 6 mbar were very slow, and the 6 mbar measurements were repeated with 0.5 K/min and longer isothermal steps at the high and low temperatures. The onset temperatures are shown in Figure 1. The onsets of the composites’ water uptake coincide with the onsets of the water uptake by pure CuCl_2_ and the MSZs are similar. This shows that the pore diameter has a limited influence on the MSZ width, corroborating the theory described earlier. Hence, the confinement inside the SG does not affect the critical nucleus size at the MSZ boundaries. Thus, nuclei of ≤5 nm are unhindered in their growth by the pores of the SG having a *d*_avg_ ≥ 5.8 nm. The MSZ boundaries found in this work were also very similar to the ones found in the literature.^9^

### Structural Transitions

**Figure 4:**
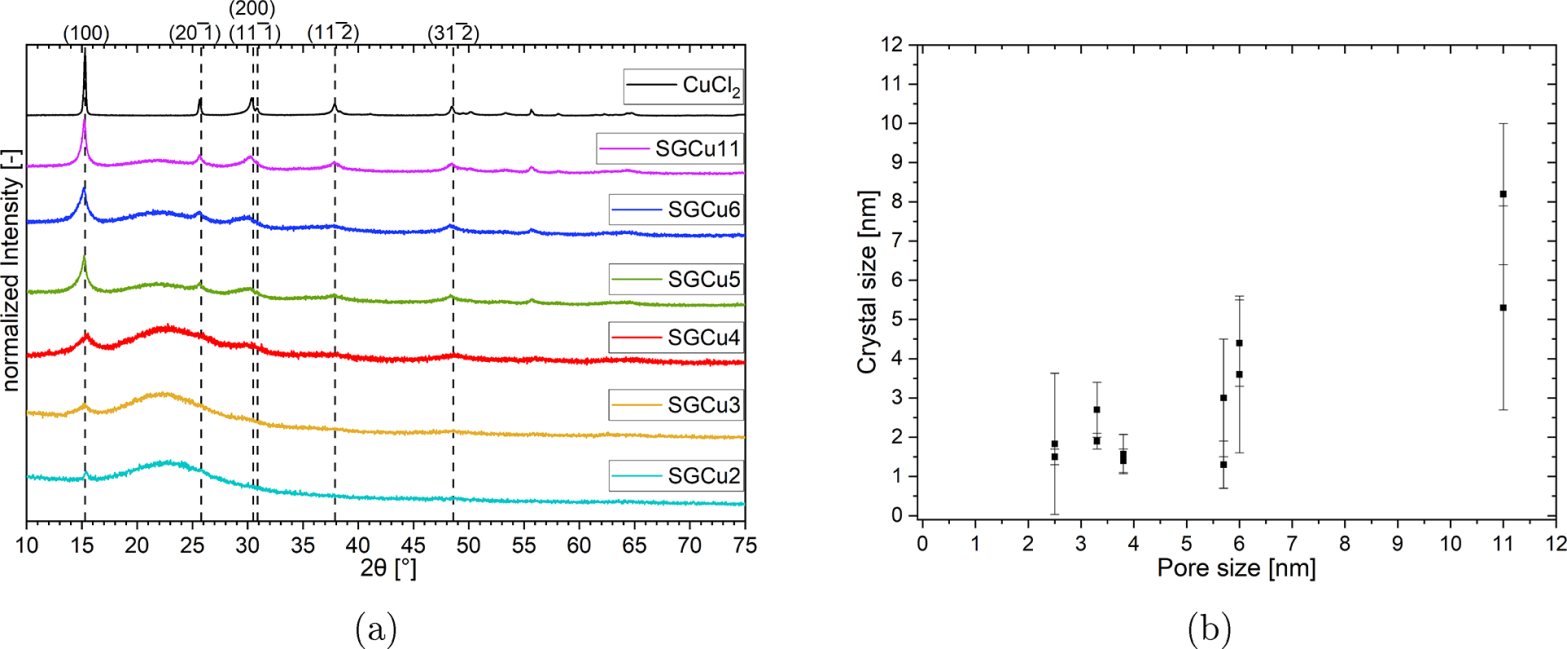
Powder-XRD measurements at 120 °C and a water vapor pressure of 0 mbar were performed on all composites and the pure bulk salt. The powder-XRD diffractograms are given in a), where the dashed vertical lines indicate the 6 highest intensity reflections of the pure anhydrous CuCl_2_ for the planes given above each line. From these diffractograms, in which each was measured twice, the average crystalline sizes of the different composite samples were estimated with the Halder-Wagner method. The results are given in b) and are plotted against the average pore size of the used silica gel in the composite.

The diffractogram of anhydrous CuCl_2_ is given in ref (53) and ref (54). The strongest reflection around 15.3° is from the (001) plane. The dihydrate CuCl_2_·2H_2_O, however, has many more reflections and stronger intensities compared to the strongest reflection, as seen in refs (55−57). The strongest one is very close to the (001) reflection of the anhydrate, at 16.2° for the (101) plane. First, a screening of all composites in an anhydrous state was performed. The results are presented in Figure 4a. For SGCu11, SGCu6, and SGCu5, the reflections of crystalline anhydrous CuCl_2_ were clearly visible and hence confirmed that crystalline salt was formed. The intensities were generally lower for the composites than for the pure salt, and the reflections were less sharp, probably due to a lower salt content in the sample and smaller crystal sizes, as smaller coherent scattering domain sizes result in lower intensities and broader reflections.

For the SGCu4, the strongest reflections attributed to CuCl_2_ were still visible but were lower in intensity, and the peaks were broader than for the other composites, indicating very small crystal sizes. The influence of the matrix was also more pronounced, as the reflections from the crystalline salt were similar in intensity to the wide amorphous reflection between 15 and 30° of the SG.

The SGCu3 and SGCu2 did not show any identifiable reflections, except around 15.3°, as seen in Figure 4a. On the one hand, the low intensities of the small pore diameter composites could be caused by the low salt content (Table 1). On the other hand, the lack of reflections other than the one around 15.3° for the smallest pore diameter composites could imply that only a small part of the impregnated CuCl_2_ was able to form crystals, while the rest remained in an amorphous form, which would not be visible in an XRD measurement. The sharp reflections for SGCu2 may indicate that a few larger CuCl_2_ crystals were formed on the outer surface of the SGs, while the salt inside the pore structure was in an amorphous form. This can be supported by the narrow full-width half maximum (fwhm) of 0.48 ± 0.1 deg of the SGCu2 reflection, which is similar to the SGCu11 composite with 0.458 ± 0.04 deg, compared to the other composites with larger fwhm’s (SGCu6: 0.848 ± 0.008, SGCu5: 0.520 ± 0.006, SGCu4: 2.32 ± 0.02, SGCu3: 2.35 ± 0.02). Additionally, the crystals on the outer surfaces could be observed with a scanning electron microscope (SEM) (see SI).

From these diffractograms, the average size of the crystallines could be estimated using the Halder-Wagner method. The results of these calculations can be found in Figure 4b. Here, it can be seen that the size of the crystallines decreases with decreasing pore diameter. Additionally, all determined average crystalline sizes are smaller than the average pore diameter, supporting the assumption that the majority of the salt hydrate is impregnated inside the pore system of the silica gel.

**Figure 5:**
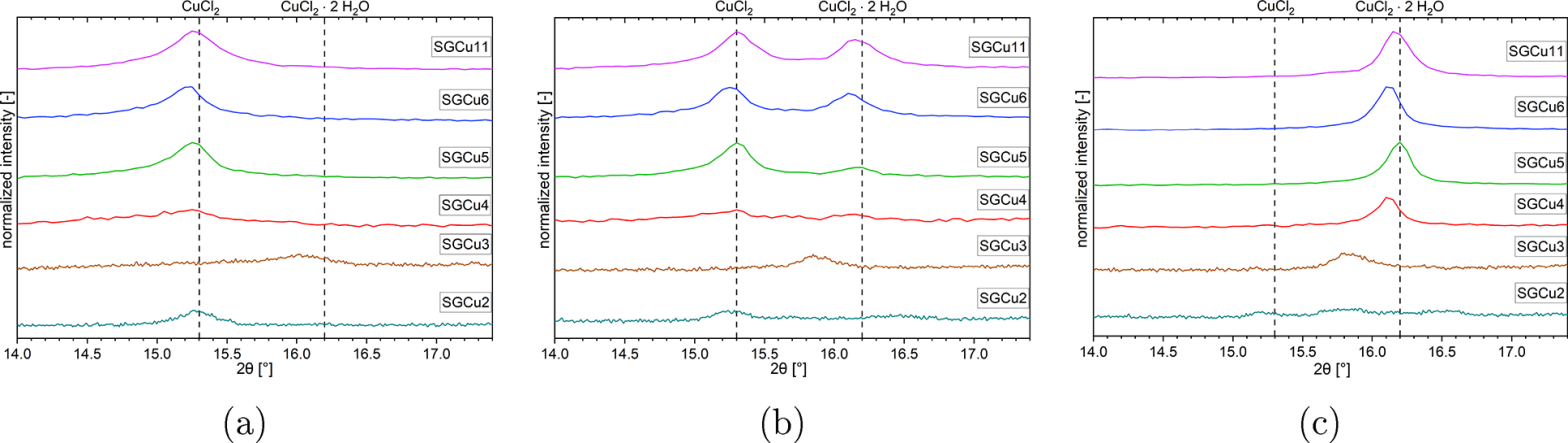
Results from isobaric PXRD in situ scans of the with CuCl_2_ impregnated silica gel composites at 10 mbar water vapor pressure and steps at temperatures of a) 120 °C, b) 36–42 °C, and c) 33 °C. The left dashed line indicates the reflection of pure anhydrous CuCl_2_ (15.3°), and the right dashed line indicates the dihydrate CuCl_2_·2H_2_O (16.2°).

As the next step, isobaric in situ measurements were performed at different water vapor pressures. The recorded diffractograms at 10 mbar for three temperatures (120 °C, 33 °C, and one between 36 and 42 °C) are shown in Figure 5. The temperature, at which a mix of both phases was visible, was different for the various samples, so a range of temperatures was chosen to depict this mixed state for all samples. At 120 °C, all composites were dehydrated, and composites showed only one reflection around the position of the anhydrous (001) plane at 15.3° (see Figure 5a). At the lowest temperature, 33 °C, the composites were completely hydrated, and the composites had only one reflection around the position of the dihydrate (101) plane at 16.2° (see Figure 5c). Between 36 and 42 °C, both phases—dihydrate and anhydrate—were detected (see Figure 5b). The position of the reflections, especially for the dihydrate, from the different composite samples deviated from the bulk salt position by up to 0.1° in 2θ. This small deviation can arise from different reasons, such as inaccuracies in the measurement, increased concentration of defects due to the smaller crystal sizes, or due to stresses on the crystals from the pore walls, which would increase for the hydrated phase since these crystals need to expand to incorporate the H_2_O molecules.

As for the pure CuCl_2_, the reflection for the dihydrate phase of the composites was more intense and sharper than for the anhydrate phase. This was most clearly visible for the composite samples with SGCu4, which had a very broad and low-intensity reflection at 120 °C with an fwhm of 1.1 ± 0.2 deg and an intensity of 8736 ± 76 counts-per-second (cps) and a stronger and sharper peak at 33 °C with an fwhm of 0.173 ± 0.013 deg and an intensity of 12634 ± 126 cps. The phase transition was also clearly visible for the composites with larger pores ( >4 nm). From this, it is clear that the SGCu4 sample had a crystalline solid–solid phase transition from the anhydrous CuCl_2_ to the dihydrate and back, even though the onsets of the transition were not distinguishable in the TGA measurements. The onsets of these phase transitions in the isobaric XRD in situ results at 10 mbar were observed around 34–38° for the hydration and around 60° for dehydration. This deviates, mainly for the dehydration, from the results obtained with the TGA experiments, which had the hydration around 36° and dehydration around 56° for the 10 mbar isobaric measurements. Reasons for this deviation are, on the one side, the difference in measurement points density. The TGA takes a measurement point every 1–9 s dependent on the total length of a single measurement, while the XRD has to stop the temperature changes every two degrees Celsius to record a diffractogram before the experiment can proceed. On the other side, the sample for the XRD in situ is much larger (95–300 mg) than the one for the TGA (4–8 mg). This leads to longer times before the sample temperature changes and to temperature gradients over the sample, which influences the phase transition of the sample in the isobaric measurements, which is sensitive to the decrease or increase in temperature. Furthermore, the uptake of water can be seen immediately in the weight changes recorded in the TGA, while in the XRD in situ measurement a certain amount of a thin surface layer of the sample needs to have changed phases to create a strong enough reflection to be recognized. This thin surface layer that is probed by the X-rays is the furthest from the temperature sensor under the sample holder in the whole sample, which leads to uncertainty between the measured sample temperature and the temperature in the probed part of the sample.

**Figure 6:**
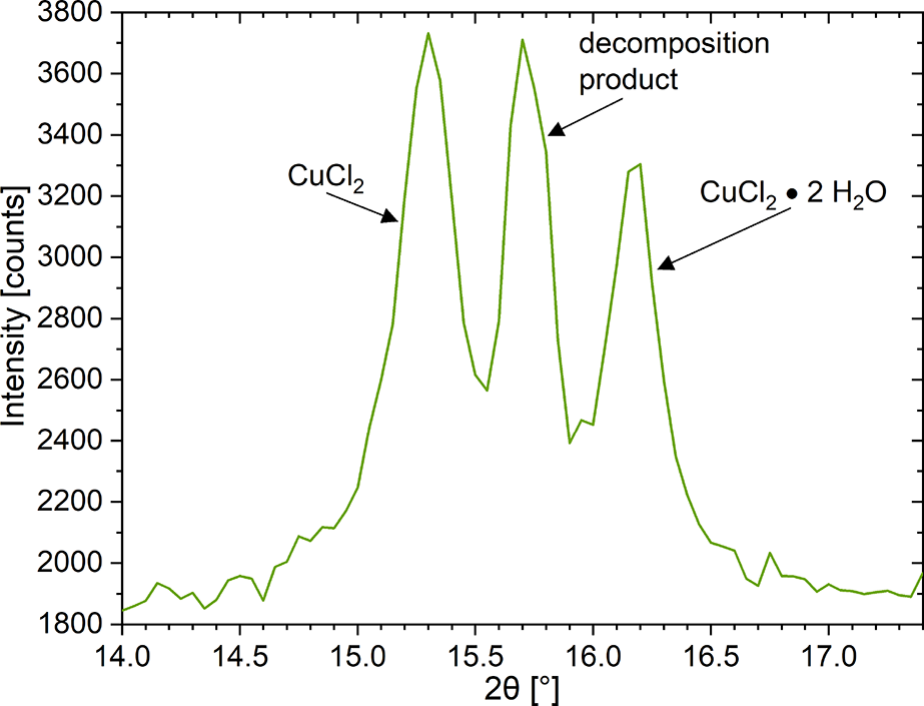
In-situ powder XRD measurement of SGCu5 at 10 mbar and temperature step 36 °C, after exposure to 12 mbar at 30 °C overnight (∼10 h). Both expected peaks of the anhydrous and dihydrate phase at 15.3° and 16.2° were visible together with a third reflection of a presumable decomposition product from hydrolysis in between.

The two smallest pore diameter samples (SGCu3 and SGCu2) showed barely any change, except for the growth of a reflection around 15.7° in between the two expected positions 15.3 and 16.2°. This may indicate a decomposition product from hydrolysis similar to MgCl_2_ in ref (58) and for CuCl_2_ in refs (59−62). This was further supported by the measurement on the SGCu5 that was deliberately exposed to 12 mbar at 30 °C for 10 h before the measurement (see Figure 6) and when a single sample was used for two consecutive in situ measurements, which gave similar results with many of the tested composites. The results clearly show a third reflection in between the reflections from the anhydrate and hydrate phases around 15.7°, which correlates with the observation from the SGCu3 and SGCu2. From this single reflection, the decomposition compound cannot be identified, but with the assumption that the only atoms/ions present were Cu^2+^, Cl^–^, H, O, and Si, the decomposition reflection could come from either CuOCuCl_2_^63−65^ or Cu_2_Cl(OH)_3_.^66^ The possible hydrolysis routes include
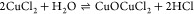
10
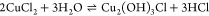
11

Additional in situ tests on the pure salt hydrate did not show this unknown reflection, which suggests that the silica gel might catalyze this hydrolysis side reaction. Hence these reflections were only observed in the composite samples and not in the pure salt ones.

### Deliquescence

In the previous sections, the solid–solid (de)hydration transition was investigated with isobaric TGA and PXRD in situ measurements. In this section, the influence of confinement on deliquescence is examined and compared to the findings in the literature with other salts.^39,40^

**Figure 7:**
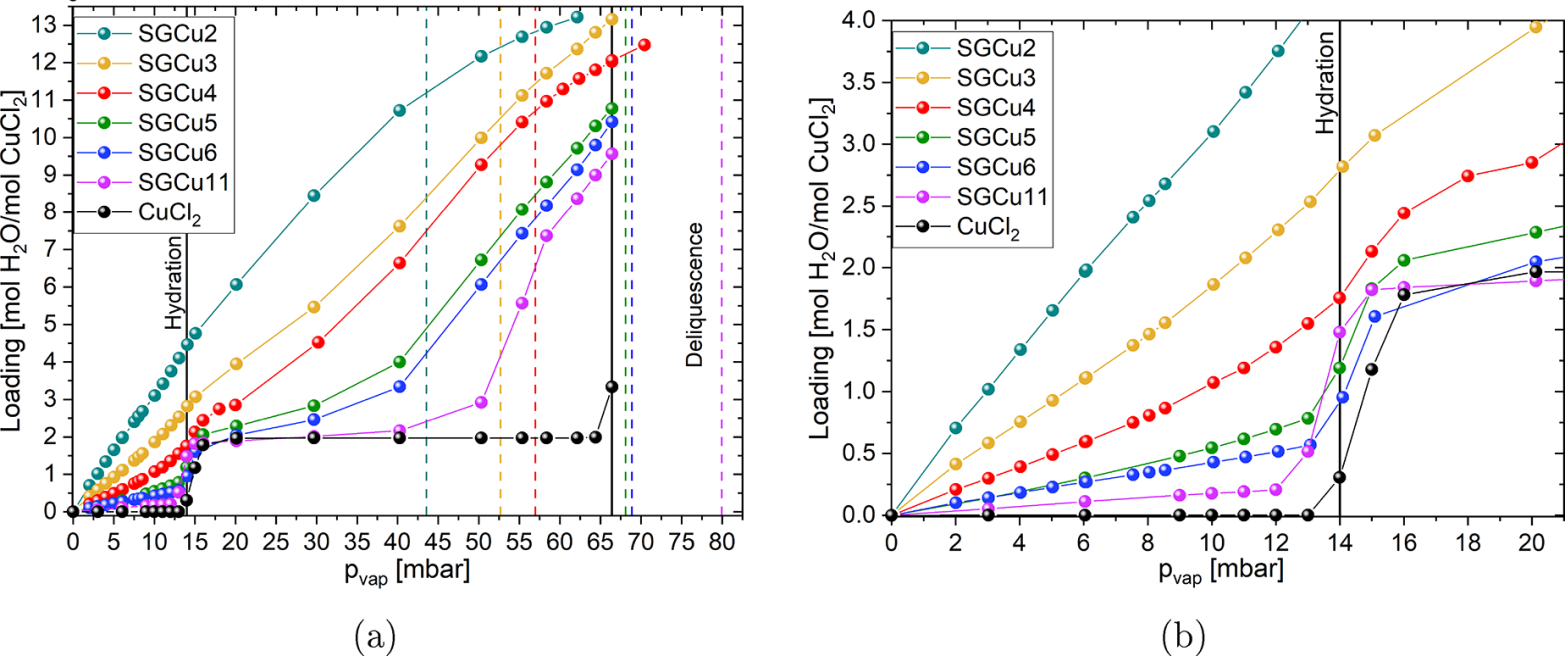
DVS was used to record the sorption isotherms at 45 °C of the tested composites and pure CuCl_2_ from anhydrate to deliquescence. The resulting data points are displayed as the loading [mol H_2_O per mol CuCl_2_] versus the water vapor pressure *p*_vap_ [mbar]. The capillary condensations of the silica gels according to the Kelvin equation are represented by the vertical lines with the corresponding color for each used pore size.

The pure salt sample does not take up water until the hydration transition around 14 mbar (Figure 7) when it absorbs an amount of water equal to 2 mol of H_2_O per mol of CuCl_2_. After this solid–solid phase transition, the weight of this sample, and hence the water content in the salt, remain constant until the deliquescence onset is around 65 mbar.

The observed hydration transition of CuCl_2_ is at a slightly lower water vapor pressure than the MSZ border determined with the isobaric TGA measurements around 17 mbar, but not at the equilibrium line of CuCl_2_ around 8 mbar.^67−69^ The small difference in hydration onsets measured between the DVS and the TGA can be explained by the long waiting times at each step in the DVS procedure (12 h, see Experimental Section), which could exceed the induction time close to the MSZ boundary.^10^ The deliquescence onset of CuCl_2_ in the literature is given as 68.1% RH at 25 °C,^67−69^ which corresponds to 65.11 mbar at 45 °C. The measured deliquescence transition conforms very well with this literature value.

The composites with larger pore diameters, SGCu11, SGCu6, SGCu5, and SGCu4, have their hydration transition around the same water vapor pressure as the pure salt. For the composites with smaller pore sizes, SGCu3 and SGCu2, no clear phase transition is observed. The unchanged hydration onset for different pore-sized samples and the absence of onsets for the two composites with the smallest pore diameters are identical to the TGA and PXRD in situ measurements. An additional similarity with the earlier measurements was the continuous adsorption from the SGs before and after the solid–solid phase transition of the salt.

In Figure 7a, it can be seen that SGCu11 has a strong water uptake above 50 mbar, which is at lower water vapor pressure than the deliquescence of the bulk salt. Furthermore, the capillary condensation of the SG11 was calculated and measured to occur at 80 mbar (see SI). From the N_2_ isotherm measurements and their analysis, it was found that the composites had a similar or slightly larger average pore diameter (*d*_avg_) than the corresponding pure SG. Therefore, the calculated and measured capillary condensation onset from the pure SGs were used. The other composites have a similar behavior with a higher uptake of water into the sample at lower water vapor pressure than the bulk salt deliquescence onset and the capillary condensation of the SG. Additionally, the higher uptake happened at lower water vapor pressures with decreasing pore size. So, the SGCu6 and SGCu5 had the stronger uptake above 40 mbar and SGCu4 above 20 mbar. For the SGCu3 and SGCu2, again no such onsets were found. In the Theory section, an estimation of the changes to the deliquescence onset of the confined salt was made and compared to the literature. By doing so, a trend of deliquescence at lower water vapor pressure for smaller pores and crystal sizes was found. Hence, the increased uptake at lower water vapor pressure with decreasing pore diameter was attributed to a shift of deliquescence to lower water vapor pressure, as predicted by eq 7 and in the literature,.^39,40^

From the curves in Figure 7, it is visible that the hydration and deliquescence transitions are nearly overlapping for the SGCu4. By following the observed trend for the deliquescence of lower water vapor pressure for smaller pore sizes, the two transitions would overlap then for SGCu3 and even more so for SGCu2. This overlap of the two transitions together with the low salt content and effects of the SGs could explain the smoothness of TGA curves and lack of dihydrate peaks found in the isobaric PXRD in situ measurements for SGCu3 and SGCu2.

## Conclusions

A series of CuCl_2_–SG composites made by impregnating CuCl_2_ into silica gels (SG) with pore diameters of 11.0, 6.0, 5.8, 3.8, 3.3, and 2.5 nm (called SGCu11, SGCu6, SGCu5, SGCu4, SGCu3, and SGCu2) were prepared and characterized with a series of physicochemical methods (TGA, in situ PXRD, DVS, N2 sorption) to study the effect of confinement on the phase transitions behavior (crystal–crystal transition, metastability, deliquescence).

In this way, no strong influences of the change in pore size and hence crystal size from the different composites on the MSZ boundaries were found. The isobaric TGA and PXRD in situ measurements returned similar results for the pure CuCl_2_ salt and the tested composites. This correlates well with the expected trends from the thermodynamics calculations in the theory section. Since the (de)hydration onset points were similar between the pure CuCl_2_ salt and the composites with visible onsets (SGCu11, SGCu6, and SGCu5) and phase transitions (also SGCu4), it can be assumed that the nuclei formation was not hindered at the MSZ boundaries, and thus these nuclei must be smaller than the confining pore diameters. All measured hydration onsets were observed below 50 °C with pore sizes of *d*_avg_ > 3.5 nm, hence according to the thermodynamic calculations the absolute values of Δγ_c_ of the salt crystal should be below 0.060 J/m^2^ for the observations of an unchanged MSZ.

Contrary to the hydration phase transition, the deliquescence onset was significantly shifted to lower water vapor pressures (*p*_vap_) with decreasing pore size. This is visible in the results from the isothermal DVS measurement and supported by the theoretical assumptions made and the findings of similar studies in the literature. The composites had stronger water uptake below the expected water vapor pressure of the deliquescence onset of the bulk salt and also below the capillary condensation of the corresponding SG. This shift to the lower water vapor pressure of the deliquescence can explain the lack of onsets found with the smallest pore-sized samples, SGCu3 and SGCu2, in regard to the (de)hydration and deliquescence transitions, as the two-phase transitions are assumed to overlap.

## Supporting Information Available

Graphic representations of the calculations done in the theory section, characterization of the used silica gels in the form of the N_2_ physisorptions with analysis, the DVS isotherms at 45 °C and isobaric TGA cycles at 10 and 20 mbar water vapor pressure. Additionally, SEM images of the composites SGCu2-SGCu4 with SEM images of multiple impregnated samples (PDF)
