# Supplementary material for: The Effect of Nanoconfinement on Deliquescence of CuCl2 Is Stronger than on Hydration
Source: Cryst Growth Des. 2023 Feb 10;23(3):1343–54. doi: 10.1021/acs.cgd.2c00821 (PMC9983011; doi:10.1021/acs.cgd.2c00821)
Supplement: Supplementary file 1 — cg2c00821_si_001.pdf [file cg2c00821_si_001.pdf]

Supporting Information for: The effect of Nano-Confinement on Deliquescence of $CuCl_2$ is stronger than on Hydration

Michaela C. Eberbach,[†,‡] Henk P. Huinink,[*,†,‡] Aleksandr I. Shkatulov,[†,¶] Hartmut R. Fischer,[§] and Olaf C.G. Adan[†,§,‖]

†*Eindhoven University of Technology, Den Dolech 2, 5600 MB Eindhoven, The Netherlands*

‡*EIRES, Horsten 1, 5612 AX Eindhoven, The Netherlands*

¶*German Aerospace Center (DLR), Pfaffenwaldring 38-40, 70569 Stuttgart, Germany*

§*TNO Materials Solutions, High Tech Campus 25, 5656 AE Eindhoven, The Netherlands*

‖*Cellcius BV, Horsten 1, 5612 AX Eindhoven, The Netherlands*

E-mail: h.p.huinink@tue.nl

Supporting Information Available

Theory Figures

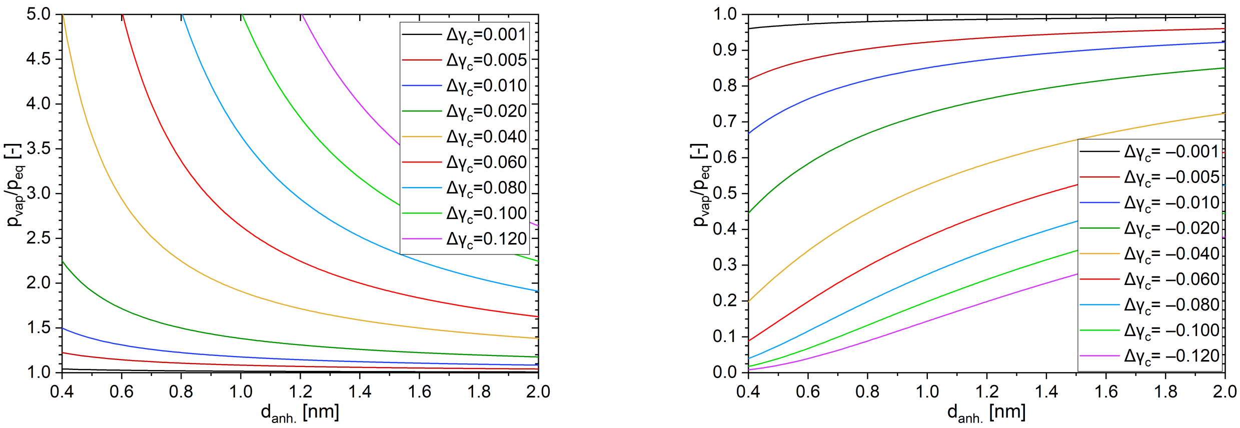

(a) (b)

Figure 1: The ratio of the water vapor pressure at the thermodynamic hydration transition point of small salt crystals with the equilibrium water vapor pressure of the bulk salt (p_{vap}/p_{eq}) as a function of the size of the spherical crystal described by the diameter of the anhydrous crystal $d_{anh.}$. This relation was calculated for differences in interfacial surface energy $\Delta\gamma_c$ of the solid salt crystal with the surrounding vapor phase. a) positive values 0.001 - 0.120 J/m²[9] and b) their negative counterparts.

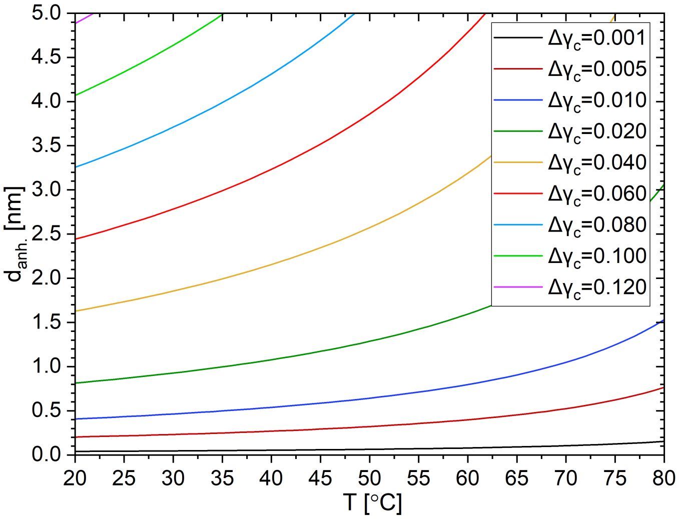

Figure 2: The relation between the critical nucleus size described by its diameter $d_{anh.}$ at the hydration MSZ boundary and the temperature T are given for different values of the specific surface/interface energy $\Delta\gamma_c$ in [J/m^2] of the $CuCl_2$ salt crystals with the vapor phase.

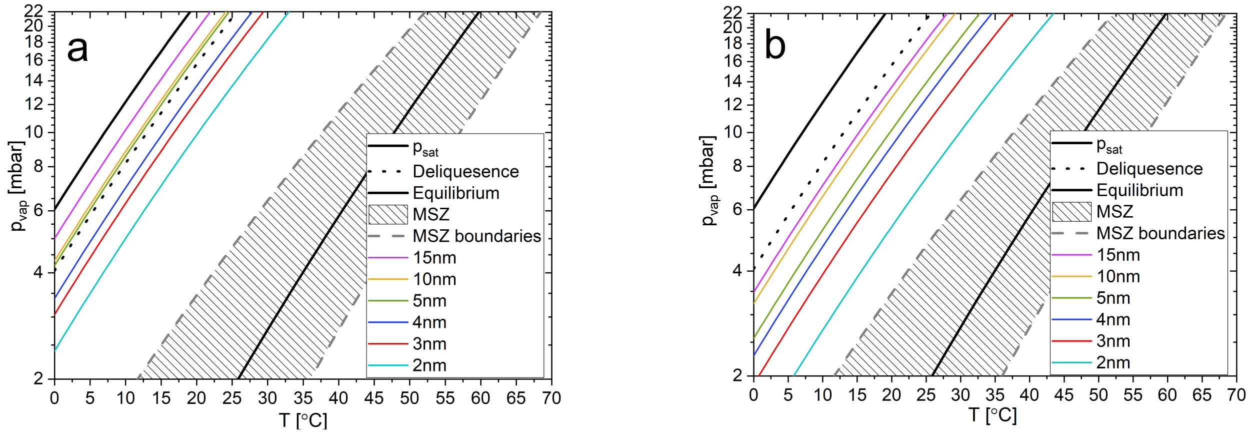

(a) (b)

Figure 3: The capillary condensation of pure water (a) and deliquescence onset of the salt (b) inside cylindrical pores as a function of pore diameter are shown calculated with the Kelvin equation. These calculations have been done with a surface tension $\gamma_{lg} = 0.072$ J/m^2, molecular volume $\upsilon = 1.80695{\cdot}10^{-5}$ m^3/mol and a contact angle $\theta = 0°$.

Characterization of porous silica gels

N_2 physisorption isotherms

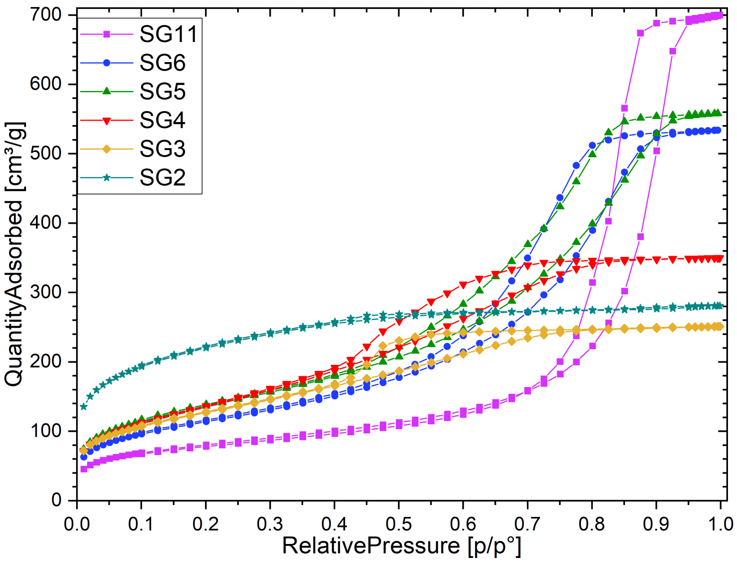

Figure 4: a) The N_2 adsorption and desorption isotherms of the different silica gels at 77 K.

Pore Structure of the silica gels

The silica gels were characterized with the N_2 adsorption and desorption isotherms using the BET method. The results for the average pore diameter d_{avg} and the pore volume V_P are given in Table 1. The V_P of the different silica gels were then used to determine the amount of $CuCl_2$ solution needed for the impregnation, as explained in the article. The d_{avg} instead was used to predict the capillary condensation with the Kelvin equation. For comparison purposes, the same characterization was done with the composites, which is shown in Table 2.

Table 1: The tested silica gels (SG) are given with the measured average pore diameters (d_{avg}) and pore volume (V_P), using N_2 gas adsorption at 77 K, together with the calculated capillary condensation onsets at 45°C in [mbar] water vapor pressure and at 20 mbar in [° C] from the Kelvin equation. Here, the values $\gamma = 0.072$ J/m^2, $\theta = 0°$ and $v = 1.80695*10^{-5}$ m^3/mol were used for the surface tension, contact angle and molar volume of water.

Silica gel name	d_{avg} [nm]	V_P [m^3/g]	capillary condensation at 45°C [mbar]	capillary condensation at 20 mbar [°C]
SG11	11.0	1.042	79.92	21
SG6	6.0	0.792	68.88	23
SG5	5.8	0.845	67.90	24
SG4	3.8	0.538	56.74	27
SG3	3.3	0.386	52.67	28
SG2	2.5	0.434	42.83	32

Silica gel sorption isotherms

As a next step, the water adsorption and desorption isotherms of the SGs were investigated. The isotherms were measured at 45°C. The results are shown in Figure 5a. All SGs have a hysteresis due to the capillary condensation in their mesopores, which happens at higher humidities than the subsequent evaporation of the water. As expected from the Kelvin equation, the capillary condensation starts at lower water vapor pressure for smaller pore sizes. So, SG2 has the lowest and SG11 the highest capillary condensation starting point. As

Table 2: The made silica gels-CuCle composites (SGCu) are given with the measured average pore diameters (d_{avg}) and pore volume (V_P), using N_2 gas adsorption at 77 K, together with the calculated capillary condensation onsets at 45°C in [mbar] water vapor pressure and at 20 mbar in [° C] from the Kelvin equation. Here, the values $\gamma = 0.072$ J/m^2, $\theta = 0°$ and $v = 1.80695*10^{-5}$ m^3/mol were used for the surface tension, contact angle and molar volume of water.

Composite name	d_{avg} [nm]	V_P [m^3/g]	capillary condensation at 45°C [mbar]	capillary condensation at 20 mbar [°C]
SGCu11	12.06	0.624	81.22	20
SGCu6	6.81	0.549	71.65	23
SGCu5	6.23	0.537	69.75	23
SGCu4	3.94	0.365	58.03	26
SGCu3	3.33	0.290	53.05	28
SGCu2	2.47	0.318	43.25	31

characterizations of the capillary condensation, the boundary points of the hystereses at lower and higher water vapor pressure were chosen to compare to the theoretical values. However, the calculated values do not overlap exactly with the measured hysteresis boundaries due to the SGs not having a single pore size but a pore size distribution with smaller and larger pores than the determined average pore size. Additionally, the hysteresis boundaries and size are pore size dependent, the maximum uptake of the SGs increases roughly with increasing pore size, which corresponds to the pore volume determined in section Pore Structure of the silica gels here above. These results show that the SGs have continuous adsorption without steps or onsets, only at high water vapor pressure does the uptake increase strongly due to capillary condensation.

The hysteresis boundaries of the different silica gels have been fitted with the Kelvin equation, lines in Figure 5b. This fit results in a contact angle $\theta = 6.07°$ and $53.21°$, based on the lower and upper points of the hysteresis, respectively. From this and the Kelvin equation, it can be seen, that the water has a small contact angle with the silica gel pore walls, which justifies the estimation of the capillary condensation with the Kelvin equation under the assumption of a 0° contact angle.

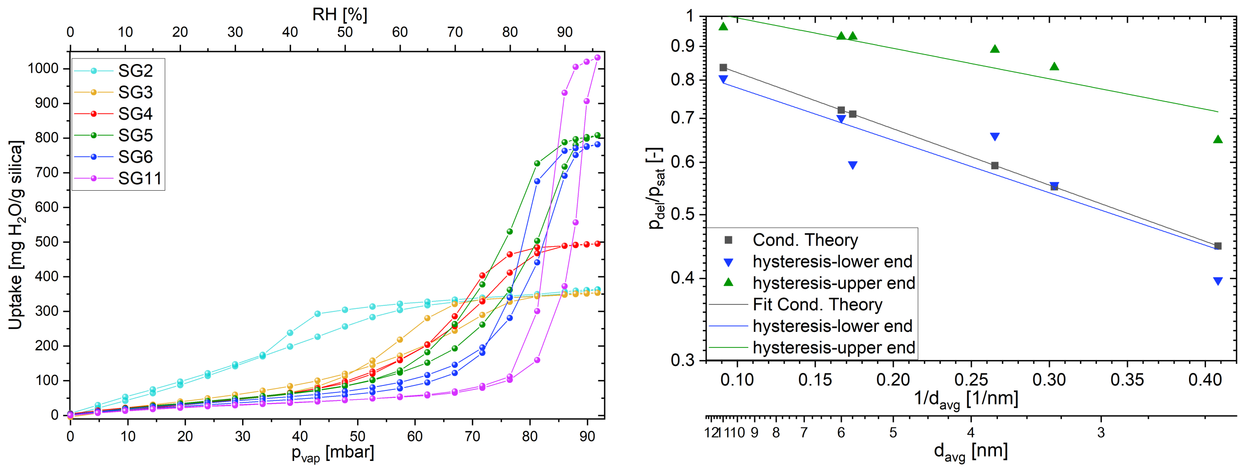

(a) (b)

Figure 5: a) The adsorption and desorption isotherms of silica gels with different pore diameters at 45°C and set humidities of 0 to 96 %RH with a maximum step size of 12 hours. The amount of sorbed water is represented in uptake [g H_2O/g silica gel] versus the set water vapor pressure (p_{vap}) and corresponding relative humidity (RH). b) The measured hysteresis boundaries of the silica gels in comparison to the results from the Kelvin equation.

Sorption behavior of silica gel under isobaric conditions

As a last step in the characterization of the SGs, the adsorption and desorption isobars were investigated. The samples were subjected to a single cycle at 10 mbar (20 mbar) water vapor pressure. A run started at 125°C (150°C). After the sample was equilibrated the temperature was ramped down to 25°C with a rate of 1 K/min (0.2 K/min) and stayed at 25°C till the weight remained constant. Finally, the temperature was ramped up to 125°C (150°C). As can be seen in Figure 6a, the SG shows a similar continuous sorption behavior as with the isothermal measurements, but without hystereses. The small difference between adsorption and desorption can be attributed to the fast ramping speed, the isothermal step at the lowest temperature, and the drift in the TGA machine. In Figure 6b, where 20 mbar water vapor pressure was used, hystereses are visible for all SGs, except for the SG11 one, which gave a similar result to the lower water vapor measurement. These hystereses correlated, like the ones from the isothermal measurements, with the trend expected from the Kelvin equation with the earliest capillary condensation in the SG2 and the latest/not visible one for the

SG11.

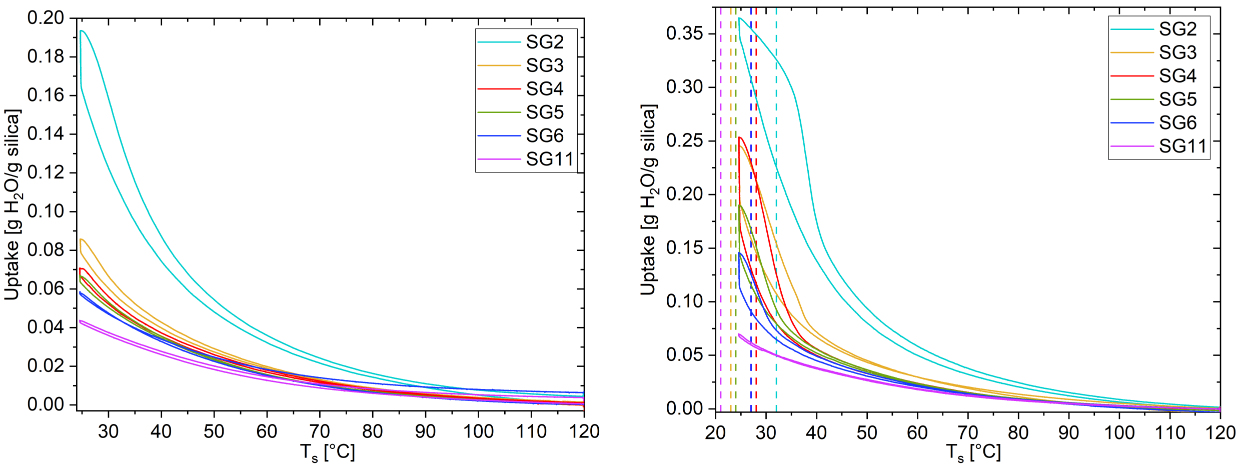

(a) 10 mbar isobar

(b) 20 mbar isobar

Figure 6: Isobaric TGA measurements at a) 10 mbar water vapor pressure with temperatures 125 to 25°C and speed 1 K/min and b) 20 mbar with 150 to 25°C with a speed of 0.2 K/min were performed. The amount of sorbed water in uptake [mg H_2O/g silica gel] is shown versus the sample temperature T_s for silica gels with different pore diameters. The capillary condensation according to the Kelvin equation is represented by the vertical lines for each used pore size.

SEM images of Composite Samples

Here, SEM images of pure silica gels and some composites are used in this article. Additionally, SEM images of multiple impregnation samples are shown to indicate the problems with them.

References

(1) Our World in Data: Global primary energy consumption by source, *https://ourworldindata.org/energy-production-consumption* (accessed 25.02.2022).

(2) ICAX: Heat Recycling from summer to winter, Sustainable Heating Sys-

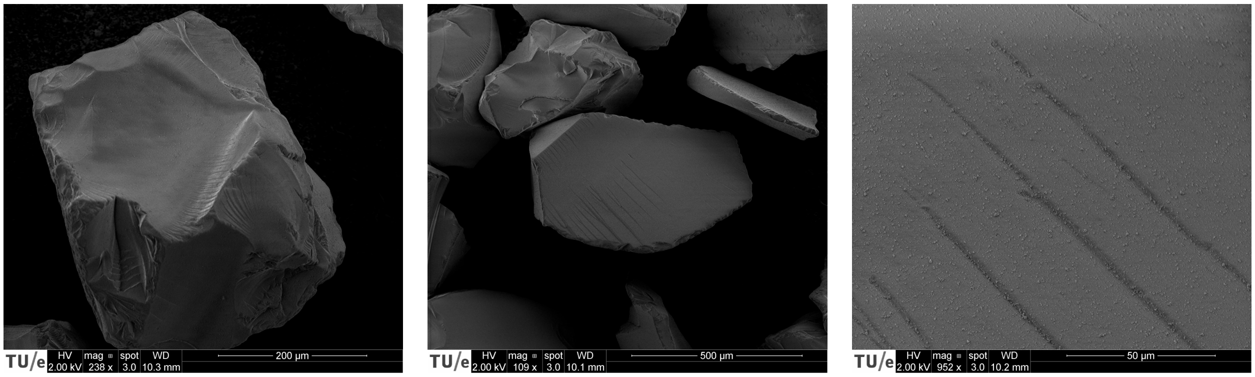

(a) (b) (c)

Figure 7: The SEM images of pure SG4 in different magnifications. This shows that pure silica gel particles have a relatively smooth outer surface with only one visible morphology and no additional growth on the surfaces.

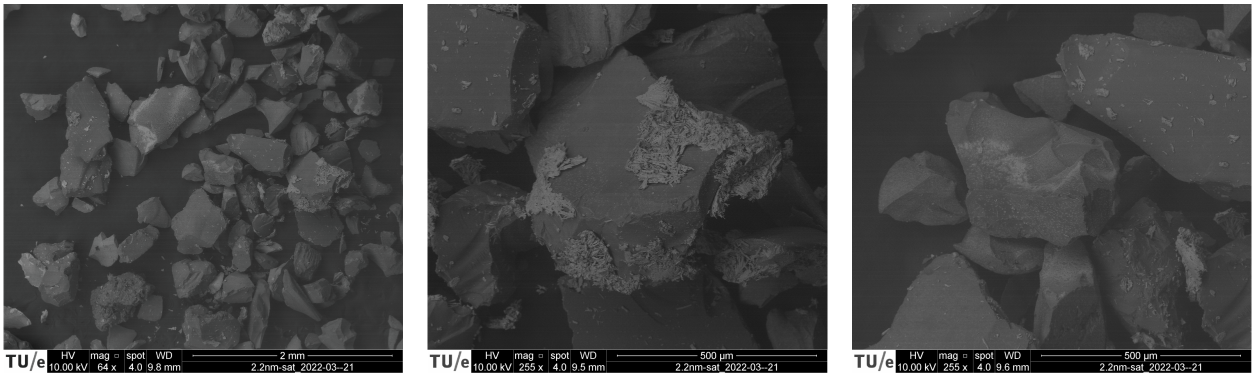

(a) (b) (c)

Figure 8: The SEM images of SGCu2 in different magnifications. This indicates that some salt crystals (bright colored) are formed on the outer surface of the silica gel particles (dark colored). However, many silica gel surfaces remain smooth without salt crystals.

tems powered by solar energy, Seasonal Thermal Energy Storage GSHC, *https://www.icax.co.uk/Heat_Recycling.html* (accessed 12.11.2020).

(3) IEA: Shares of residential energy consumption by end use in selected IEA countries in 2018, *https://www.iea.org/data-and-statistics/charts/shares-of-residential-energy-consumption-by-end-use-in-selected-iea-countries-2018* (accessed 18.03.2021).

(4) P. Tatsidjodoung, N. Le Pierrés and L. Luo, A review of potential materials for thermal

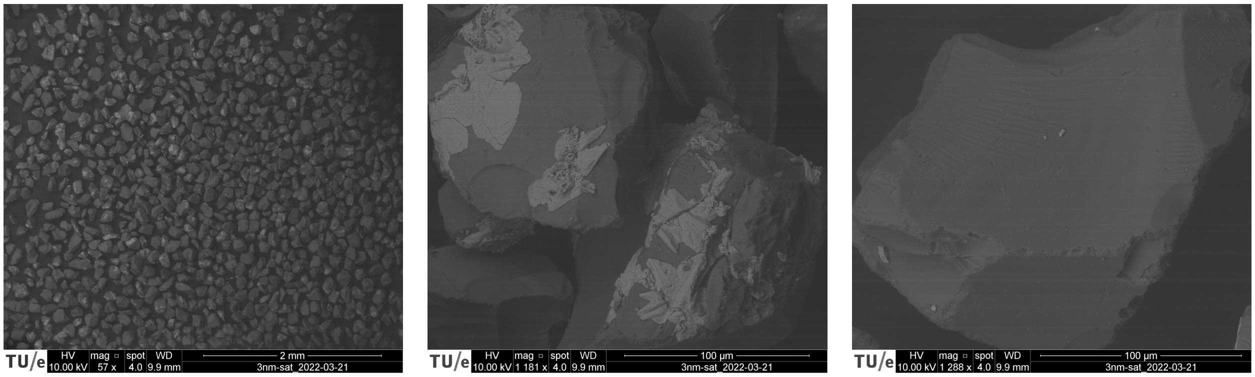

(a) (b) (c)

Figure 9: The SEM images of SGCu3 in different magnifications. This indicates that some salt crystals (bright colored) are formed on the outer surface of the silica gel particles (dark colored). However, many silica gel surfaces remain smooth without salt crystals.

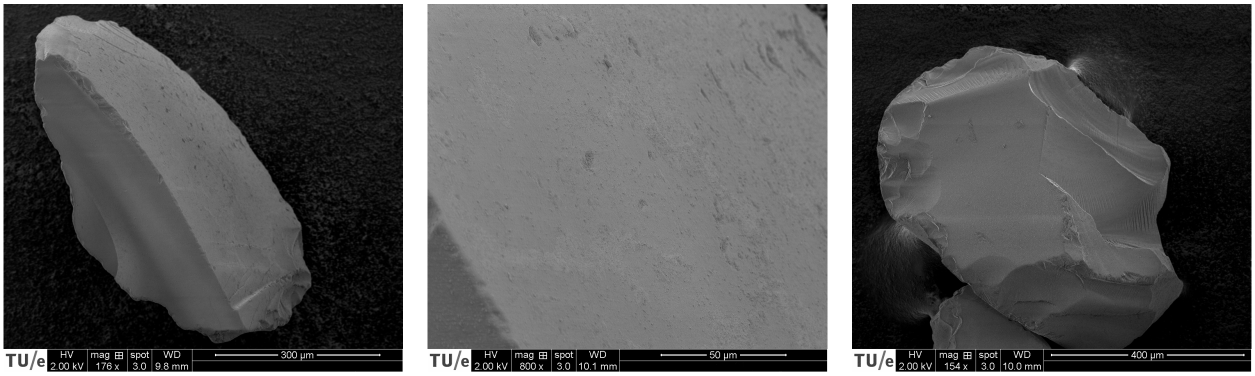

(a) (b) (c)

Figure 10: The SEM images of SGCu4 in different magnifications. On these silica gel particles are no salt crystals or salt formations visible, indicating that (nearly) all $CuCl_2$ was impregnated into the pore systems.

energy storage in building applications, *Elsevier Renewable and Sustainable Energy Reviews* **2012**, Volume 18, p. 327–349. DOI: http://dx.doi.org/10.1016/j.rser.2012.10.025

(5) L. Scapino, H.A. Zondag, J. Van Bael, J. Diriken and C.C.M. Rindt, Sorption heat storage for long-term low-temperature applications: A review on the advancements at material and prototype scale, *Elsevier Applied Energy* **2017**, Volume 190, pp. 920-948. DOI: https://doi.org/10.1016/j.apenergy.2016.12.148

(6) Y. Kato, Chemical energy conversion technologies for efficient energy use, *Springer*

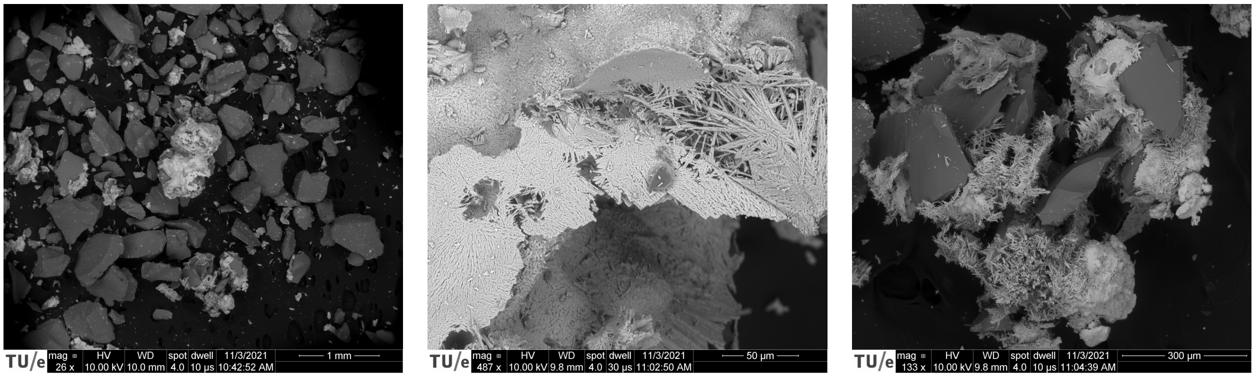

(a) (b) (c)

Figure 11: The SEM images of SGCu2, which were impregnated twice, in different magnifications. On these silica gel particles are many larger salt crystals with the needle morphology, characteristic for $CuCl_2$, visible, and some particles are connected by these salt crystals. However, there are still many silica gel particles with little salt formations on their outer surfaces.

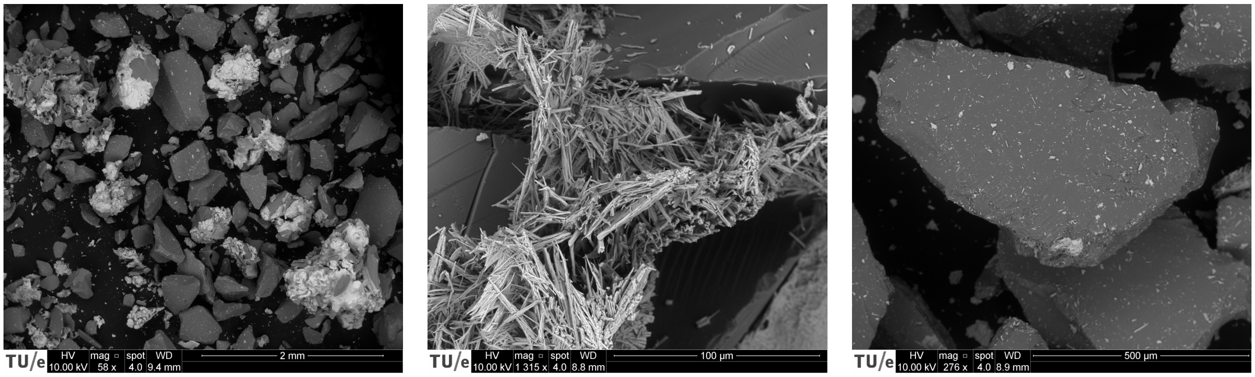

(a) (b) (c)

Figure 12: The SEM images of SGCu2, which were impregnated three times, in different magnifications. On these silica gel particles are many larger salt crystals with the needle morphology, characteristic for $CuCl_2$, visible and several particles are connected by these salt crystals. Only some silica gel particles remain with little salt formations on their outer surfaces.

Link Thermal Energy Storage for Sustainable Energy Consumption **2007**, NATO Science Series (Mathematics, Physics and Chemistry), Vol 234, pp. 377-391. DOI: https://doi.org/10.1007/978-1-4020-5290-3_23

(7) D. Dicaire and F. H. Tezel, Regeneration and efficiency characterization of hybrid adsorbent for Thermal Energy Storage of excess and solar heat, *Elsevier Renewable Energy*

2010, Volume 36, pp. 986-992. DOI: https://doi.org/10.1016/j.renene.2010.08.031

(8) J. Jänchen, D. Ackermann, H. Stach and W. Brösicke, Studies of the water adsorption on zeolites and modified mesoporous materials for seasonal storage of solar heat, *Elsevier Solar energy* **2003**, Volume 76, pp. 339-344. DOI: https://doi.org/10.1016/j.solener.2003.07.036

(9) L.-C. Sögütoglu, M. Steiger, J. Houben, D. Biemans, H.R. Fischer, P. Donkers, H.P. Huinink and O.C.G. Adan, Understanding the Hydration Process of Salts: The Impact of a Nucleation Barrier, *American Chemical Society Crystal Growth & Design* **2019**, Volume 19, pp. 2279-2288. DOI: https://doi.org/10.1021/acs.cgd.8b01908

(10) L.-C. Sögütoglu, F. Birkelbach, A. Werner, H.R. Fischer, H.P. Huinink and O.C.G. Adan, Hydration of salts as a two-step process: Water adsorption and hydrate formation, *Elsevier Thermochimica Acta* **2021**, Volume 695, 178819. DOI: https://doi.org/10.1016/j.tca.2020.178819

(11) K. Linnow, M. Niermann, D. Bonatz, K. Posern and M. Steiger, Experimental studies of the mechanism and kinetics of hydration reactions, SHC 2013, International Conference on Solar Heating and Cooling for Buildings and Industry September 23-25, 2013, Freiburg, Germany, *Elsevier Energy Procedia* **2014**, Volume 48, pp. 394-404. DOI: 10.1016/j.egypro.2014.02.046

(12) K.E. N'Tsoukpoe, H. Liu, N. Le Pierrès, L. Luo, A review on long-term sorption solar energy storage, *Elsevier Renewable and Sustainable Energy Reviews* **2009**, Volume 13, pp. 2385–2396. DOI: https://doi.org/10.1016/j.rser.2009.05.008

(13) N. Yu, R.Z. Wang, L.W. Wang, Review - Sorption thermal storage for solar energy, *Elsevier Progress in Energy and Combustion Science* **2013**, Volume 39, pp. 489-514. DOI: https://doi.org/10.1016/j.pecs.2013.05.004

(14) R.-J. Clark, A. Mehrabadi, M. Farid, State of the art on salt hydrate thermochemical energy storage systems for use in building applications, *Elsevier Journal of Energy Storage* **2020**, Volume 27, 101145. DOI: https://doi.org/10.1016/j.est.2019.101145

(15) H. Liu, K. Nagano and J. Togawa, A composite material made of mesoporous siliceous shale impregnated with lithium chloride for an open sorption thermal energy storage system, *Elsevier Solar Energy* **2015**, Volume 111, pp. 186-200. DOI: http://dx.doi.org/10.1016/j.solener.2014.10.044

(16) Yu. I. Aristov, New Composite Adsorbents for Conversion and Storage of Low-Temperature Heat: Activity in the Boreskov Institute of Catalysis, *Journal of the Heat Transfer Society of Japan* **2006**, Volume 45, pp. 12-19. DOI: https://doi.org/10.11368/htsj1999.45.192_12

(17) L. G. Gordeeva and Yu. I. Aristov, Composites 'salt inside porous matrix' for adsorption heat transformation: a current state-of-the-art and new trends, *International Journal of Low-Carbon Technologies* **2012**, Volume 7, pp. 288–302. DOI: https://doi.org/10.1093/ijlct/cts050

(18) Yu. I. Aristov, *Nanocomposite Sorbents for Multiple Applications*, **2020**, Jenny Stanford Publishing Pte. Ltd., ISBN 978-981-4267-50-2 (Hardcover), ISBN 978-981-4303-15-6 (eBook).

(19) Yu. I. Aristov, Novel Materials for Adsorption Heat Pumping and Storage: Screening and Nanotailoring of Sorption Properties, *Journal of Chemical Engineering of Japan* **2007**, Volume 40, pp. 1242-1251. DOI: https://doi.org/10.1252/jcej.07WE228

(20) Yu. Pankratév, M.M. Tokarev and Yu I. Aristov, Heats of water sorption on silica gel containing $CaCl_2$ and LiBr, *Russian Journal of Physical Chemistry A* **2001**, Volume 75, pp. 806-810.

(21) A. Jabbari-Hichri, S. Bennici, and A. Auroux, Effect of aluminum sulfate addition on the thermal storage performance of mesoporous SBA-15 and MCM-41 materials, *Elsevier Solar Energy Materials & Solar Cells* **2016**, Volume 149, pp. 232-241. DOI: http://dx.doi.org/10.1016/j.solmat.2016.01.033

(22) S. Hongois, F. Kuznik, P. Stevens and J.-J. Roux, Development and characterization of a new MgSO4-zeolite composite for long-term thermal energy storage, *Elsevier Solar Energy Materials & SolarCells* **2011**, Volume 95, pp. 1831-1837. DOI: 10.1016/j.solmat.2011.01.050

(23) T. Nonnen, H Preißler, S. Kött, S. Beckert and R. Gläser, Salt inclusion and deliquescence in salt/zeolite X composites for thermochemical heat storage, *Elsevier Microporous and Mesoporous Materials* **2020**, Volume 303, 110239. DOI: https://doi.org/10.1016/j.micromeso.2020.110239

(24) Yu. I. Aristov, New Family of Solid Sorbents for Adsorptive Cooling: Material Scientist Approach, *Springer Link Journal of Engineering Thermophysics* **2007**, Volume 16, pp. 63–72. DOI: https://doi.org/10.1134/S1810232807020026

(25) A.I. Shkatulov, J. Houben, H. Fischer and H.P. Huinink, Stabilization of K_2CO_3 in vermiculite for thermochemical energy storage, *Elsevier Renewable Energy* **2020**, Volume 150, pp. 990-1000. DOI: https://doi.org/10.1016/j.renene.2019.11.119

(26) A.I. Shkatulov, R. Joosten, H. Fischer and H.P. Huinink, Core-Shell Encapsulation of Salt Hydrates into Mesoporous Silica Shells for Thermochemical Energy Storage, *American Chemistry Society Applied Energy Matter* **2020**, Volume 3, pp. 6860-6869. DOI: https://doi.org/10.1021/acsaem.0c00971

(27) A. Permyakova, S. Wang, E. Courbon, F. Nouar, N. Heymans, P. D'Ans, N. Barrier, P. Billemont, G. De Weireld, N. Steunou, M. Frère and C. Serre, Design of salt-metal organic framework composites for seasonal heat storage applications, *The Royal Society*

of Chemistry Journal of Materials Chemistry A **2017**, Volume 5, pp. 12889-12898. DOI: https://doi.org/10.1039/C7TA03069J

(28) K. Posern, K. Linnow, M. Niermann, Ch. Kaps and M. Steiger, Thermochemical investigation of the water uptake behavior of $MgSO_4$ hydrates in host materials with different pore size, *Elsevier Thermochimica Acta* **2015**, Volume 611, pp. 1-9. DOI: http://dx.doi.org/10.1016/j.tca.2015.04.031

(29) E. Courbon, P. D'Ans, A. Permyakova, O. Skrylnyk, N. Steunou, M. Degrez and M. Frère, A new composite sorbent based on $SrBr_2$ and silica gel for solar energy storage application with high energy storage density and stability, *Elsevier Applied Energy* **2017**, Volume 190, pp. 1184-1194. DOI: http://dx.doi.org/10.1016/j.apenergy.2017.01.041

(30) P. D'Ans, E. Courbon, A. Permyakova, F. Nouar, C. Simonnet-Jégat, F. Bourdreux, L. Malet, C. Serre, M. Frère and N. Steunou, A new Strontium Bromide MOF Composite with Improved Performance for Solar Energy Storage Applications, *Elsevier The Journal of Energy Storage* **2019**, Volume 25, 100881. DOI: https://doi.org/10.1016/j.est.2019.100881

(31) P.A. Kallenberger, K. Posern, K. Linnow, F.J. Brieler, M. Steiger and M. Fröba, Alginate-Derived Salt/Polymer Composites for Thermochemical Heat Storage, *Advanced Sustainable Systems* **2018**, Volume 2, 1700160. DOI: 10.1002/adsu.201700160

(32) A. Palacios, M.E. Navarro, C. Barreneche and Y. Ding, Hybrid 3-in-1 thermal energy storage system – Outlook for a novel storage strategy, *Elsevier Applied Energy* **2020**, Volume 274, pp. 115024. DOI: https://doi.org/10.1016/j.apenergy.2020.115024

(33) V. Brancato, L. Calabrese, V. Palomba, A. Frazzica, M. Fullana-Puig, A. Solé and L.F. Cabeza, $MgSO_4 \cdot 7\,H_2O$ filled macro cellular foams: An innovative composite sorbent for thermo-chemical energy storage applications for solar buildings, *Elsevier Solar Energy* **2018**, Volume 173, pp. 1278-1286. DOI: https://doi.org/10.1016/j.solener.2018.08.075

(34) L. Calabrese, V. Brancato, V. Palomba, A. Frazzica and L.F. Cabeza, Innovative composite sorbent for thermal energy storage based on a $SrBr_2 \cdot 6\,H_2O$ filled silicone composite foam, *Elsevier Journal of Energy Storage* **2019**, Volume 26, 100954. DOI: https://doi.org/10.1016/j.est.2019.100954

(35) Yu.I. Aristov, G. Restuccia, G. Cacciola and V.N. Parmon, A family of new working materials for solid sorption air conditioning systems, *Elsevier Applied Thermal Engineering* **2002**, Volume 22, pp. 191-204, 2002. DOI: https://doi.org/10.1016/S1359-4311(01)00072-2

(36) G.T. Whiting, D. Grondin, S. Bennici and A. Auroux, Heats of water sorption studies on zeolite–$MgSO_4$ composites as potential thermochemical heat storage materials, *Elsevier Solar Energy Materials and Solar Cells* **2013**, Volume 112, pp. 112–119. DOI: https://doi.org/10.1016/j.solmat.2013.01.020

(37) I.A. Simonova and Yu.I. Aristov, Sorption Properties of Calcium Nitrate Dispersed in Silica Gel: the Effect of Pore Size, *Russian Journal of Physical Chemistry A* **2005**, Volume 79, pp. 1307-1311.

(38) P.A.J. Donkers, L. Pel and O.C.G. Adan, Experimental studies for the cyclability of salt hydrates for thermochemical heat storage, *Elsevier Journal of Energy Storage* **2015**, Volume 5, pp. 25-32. DOI: https://doi.org/10.1016/j.est.2015.11.005

(39) P. Jain, O. Vincent, and A.D. Stroock, Adsorption, Desorption, and Crystallization of Aqueous Solutions in Nanopores, *American Chemical Society Langmuir* **2019**, Volume 35, pp. 3949-3962. DOI: 10.1021/acs.langmuir.8b04307

(40) T. Talreja-Muthreja, K. Linnow, D. Enke and M. Steiger, Deliquescence of NaCl Confined in Nanoporous Silica, *American Chemical Society Langmuir* **2022**, Volume 38, pp. 10963-10974. DOI: https://doi.org/10.1021/acs.langmuir.2c01309

(41) L.-C. Sögütoglu, M. Steiger, J. Houben, D. Biemans, H.R. Fischer, P. Donkers, H.P. Huinink and O.C.G. Adan, Understanding the Hydration Process of Salts: The Impact of a Nucleation Barrier - Supporting information, *American Chemical Society Crystal Growth & Design* **2019**, Volume 19, pp. 2279-2288. DOI: 10.1021/acs.cgd.8b01908

(42) Dimo Kashchiev, *Nucleation 1st Edition*, Butterworth-Heinemann 2000, February 22, 2000, Hardcover ISBN: 9780750646826. DOI: https://doi.org/10.1016/B978-0-7506-4682-6.X5000-8

(43) B.J. Cross, *Some Capillary Condensation Studies of Pore Structure*, University of Bristol, 1969. Available on: https://books.google.nl/books?id=tRwyzgEACAAJ (accessed 07.09.2022)

(44) J. Lyklema, *Fundamentals of Interface and Colloid Science - Volume 2: Solid-Liquid Interfaces*, 1995. ISSN 1874-5679, ISBN 9780124605244, https://doi.org/10.1016/S1874-5679(06)80012-7

(45) P. Mirabel, H. Reiss, and R.K. Bowles, A theory for the deliquescence of small particles, *American Institute of Physics The Journal of Chemical Physics* **2000**, Volume 113, pp. 8200-8205. DOI: https://doi.org/10.1063/1.1315993

(46) G.T. Whiting, D. Grondin, D. Stosic, S. Bennici, and A. Auroux, Zeolite–$MgCl_2$ composites as potential long-term heat storage materials: Influence of zeolite properties on heats of water sorption, *Elsevier Solar Energy Materials and Solar Cells* **2014**, Volume 128, pp. 289–295. DOI: http://dx.doi.org/10.1016/j.solmat.2014.05.016

(47) Yu.I. Aristov, M.M. Tokarev, G. Cacciola and G. Restuccia, Selective Water Sorbents for Multiple Applications, 1. $CaCl_2$ Confined in Mesopores of Silica Gel: Sorption Properties, *Springer Link Reaction Kinetics and Catalysis Letters* **1996**, Volume 59, p. 335-342. DOI: https://doi.org/10.1007/BF02068130

(48) E.P. Barrett, L.G. Joyner, and P.P. Halenda, The Determination of Pore Volume and Area Distributions in Porous Substances. I. Computations from Nitrogen Isotherms, *American Chemistry Society Journal of the American Chemical Society* **1951**, Volume 73, pp. 373-380. DOI: https://doi.org/10.1021/ja01145a126

(49) S. Brunauer, P.H. Emmett and E. Teller, Adsorption of Gases in Multimolecular Layers, *American Chemistry Society Journal of the American Chemical Society* **1938**, Volume 60, pp. 309-319. DOI: https://doi.org/10.1021/ja01269a023

(50) P. Gabbott, *Principles and applications of thermal analysis*, John Wiley & Sons, 2008. Print ISBN:9781405131711, Online ISBN:9780470697702, DOI:10.1002/9780470697702

(51) L. Greenspan, Humidity fixed points of binary saturated aqueous solutions, *Journal of Research of the National Bureau of Standards. Section A, Physics and Chemistry* **1977**, Volume 81, pp. 89-96. DOI: 10.6028/jres.081A.011

(52) L.-C. Sögütoglu, *Fundamentals of salt hydration for heat battery application - Appendix: XRD Analysis of salt hydrates*, Ph.D. thesis, Eindhoven University of Technology, 2020. Available on: https://research.tue.nl/en/publications/fundamentals-of-salt-hydration-for-heat-battery-application (accessed 07.09.2022)

(53) A.F. Wells, 333. The crystal structure of anhydrous cupric chloride, and the stereochemistry of the cupric atom, *The Royal Society of Chemistry Journal of the Chemical Society* **1947**, pp. 1670-1675. DOI: https://doi.org/10.1039/JR9470001670

(54) P.C. Burns and F.C. Hawthorne, Tolbachite, CuCl2, the first example of Cu2+ octahedrally coordinated by Cl, *American Mineralogist* **1993**, Volume 78, pp. 187-189.

(55) C.H. MacGillavry and J.M. Bijvoet, Die Kristallstruktur der Cadmium- und QuecksilberDiammin-Dihalogenide, *Zeitschrift fuer Kristallographie, Kristallgeometrie, Kristallphysik, Kristallchemie* **1936**, Volume 94, pp. 231 - 245.

(56) D. Harker, *The Crystal Structure of Cupric Chloride Dihydrate Cu Cl2 (H2 O)2, Zeitschrift fuer Kristallographie, Kristallgeometrie, Kristallphysik, Kristallchemie* **1936**, Volume 93, pp. 136-145.

(57) A. Engberg, An X-Ray refinement of the crystal structure of copper(II) chloride dihydrate, *Acta Chemica Scandinavica* **1970**, Volume 24, pp. 3510-3526. DOI: 10.3891/acta.chem.scand.24-3510

(58) Y. Kirsh, S. Yariv, and S. Shoval, Kinetic Analysis of Thermal Dehydration and Hydrolysis of $MgCl_2 \cdot 6\,H_{20}$ by DTA and TG, *John Wiley & Sons Journal of Thermal Analysis* **1987**, Volume 32, pp. 393-408. DOI: https://doi.org/10.1007/BF01912692

(59) I. Dincer and C. Zamfirescu, Sustainable Hydrogen Production - Chapter 4 - Hydrogen Production by Thermal Energy, *Elsevier* **2016**, pp. 163-308. ISBN 9780128015636, DOI: https://doi.org/10.1016/B978-0-12-801563-6.00004-2

(60) D. Thomas, N.A. Baveja, K.T. Shenoy and J.B. Joshi, Experimental Study on the Mechanism and Kinetics of CuCl2 Hydrolysis Reaction of the Cu–Cl Thermochemical Cycle in a Fluidized Bed Reactor, *ACS Publications Industrial & Engineering Chemistry Research* **2020**, Volume 59, pp. 12028-12037. DOI: https://dx.doi.org/10.1021/acs.iecr.0c01807

(61) A. Farsi, I. Dincer and G.F. Naterer, Second law analysis of CuCl2 hydrolysis reaction in the Cu–Cl thermochemical cycle of hydrogen production, *Elsevier Energy* **2020**, Volume 202, 117721. DOI: https://doi.org/10.1016/j.energy.2020.117721

(62) D. Thomas, N.A. Baveja, K.T. Shenoy and J.B. Joshi, Mechanistic and kinetic study of thermolysis reaction with hydrolysis step products in Cu–Cl thermochemical cycle, *Elsevier International Journal of Hydrogen Energy* **2021**, Volume 46, pp. 12672-12681. DOI: https://doi.org/10.1016/j.ijhydene.2021.01.118

(63) M.A. Lewis and J.G. Masin, The evaluation of alternative thermochemical cycles – Part II: The down-selection process, *Elsevier International Journal of Hydrogen Energy* **2009**, Volume 34, pp. 4125-4135. DOI: https://doi.org/10.1016/j.ijhydene.2008.07.085

(64) M.S. Ferrandon, M.A. Lewis, F. Alvarez and E. Shafirovich, Hydrolysis of CuCl2 in the Cu–Cl thermochemical cycle for hydrogen production: Experimental studies using a spray reactor with an ultrasonic atomizer, *Elsevier International Journal of Hydrogen Energy* **2010**, Volume 35, pp. 1895-1904. DOI: https://doi.org/10.1016/j.ijhydene.2009.12.034

(65) S. Ghandehariun, M.A. Rosen, G.F. Naterer and Z. Wang, Comparison of molten salt heat recovery options in the Cu-Cl cycle of hydrogen production, *Elsevier International Journal of Hydrogen Energy* **2011**, Volume 36, pp. 11328-11337. DOI: https://doi.org/10.1016/j.ijhydene.2010.11.093

(66) C. Engelbrekt, P. Malcho, J. Andersen, L. Zhang, K. Stahl, B. Li, J. Hu and J. Zhang, Selective synthesis of clinoatacamite $Cu_2(OH)_3Cl$ and tenorite CuO nanoparticles by pH control, *Springer Link Journal of Nanoparticle Research volume* **2014**, Volume 16, pp. 2562. DOI: https://doi.org/10.1007/s11051-014-2562-4

(67) M. Richter, E.-M. Habermann, E. Siebecke, M. Linder, A systematic screening of salt hydrates as materials for thermochemical heat transformer, *Elsevier Thermochimica Acta* **2018**, Volume 659, pp. 136-150. DOI: https://doi.org/10.1016/j.tca.2017.06.011

(68) L. Glasser, Thermodynamics of inorganic hydration and of humidity control, with an extensive database of salt hydrate pairs, *American Chemical Society ournal of Chemical & Engineering Data* **2014**, Volume 59, pp. 526-530. DOI: https://doi.org/10.1021/je401077x

(69) O. Polyachenok, E. Dudkina, L. Polyachenok, Thermal stability and thermodynamics

of copper(ii) chloride dihydrate, *Elsevier The Journal of Chemical Thermodynamics* **2009**, Volume 41, pp. 74-79. DOI: https://doi.org/10.1016/j.jct.2008.07.018
